# Structurally detailed coarse-grained model for Sec-facilitated co-translational protein translocation and membrane integration

**DOI:** 10.1371/journal.pcbi.1005427

**Published:** 2017-03-22

**Authors:** Michiel J. M. Niesen, Connie Y. Wang, Reid C. Van Lehn, Thomas F. Miller

**Affiliations:** Division of Chemistry and Chemical Engineering, California Institute of Technology, Pasadena, California, United States of America; Stockholm University, SWEDEN

## Abstract

We present a coarse-grained simulation model that is capable of simulating the minute-timescale dynamics of protein translocation and membrane integration via the Sec translocon, while retaining sufficient chemical and structural detail to capture many of the sequence-specific interactions that drive these processes. The model includes accurate geometric representations of the ribosome and Sec translocon, obtained directly from experimental structures, and interactions parameterized from nearly 200 *μ*s of residue-based coarse-grained molecular dynamics simulations. A protocol for mapping amino-acid sequences to coarse-grained beads enables the direct simulation of trajectories for the co-translational insertion of arbitrary polypeptide sequences into the Sec translocon. The model reproduces experimentally observed features of membrane protein integration, including the efficiency with which polypeptide domains integrate into the membrane, the variation in integration efficiency upon single amino-acid mutations, and the orientation of transmembrane domains. The central advantage of the model is that it connects sequence-level protein features to biological observables and timescales, enabling direct simulation for the mechanistic analysis of co-translational integration and for the engineering of membrane proteins with enhanced membrane integration efficiency.

## Author summary

Ubiquitous across all kingdoms of life, the Sec translocon is an essential piece of molecular machinery for protein biosynthesis. The translocon is a transmembrane channel that enables the secretion of newly synthesized proteins across the lipid membrane, as well as the integration of protein domains into the membrane interior. Understanding the function and regulation of the translocon is necessary for developing a refined view of early stage protein folding and targeting in the cell. Although computational methods are well suited to elucidating the interactions of the translocon with newly synthesized proteins, conventional simulation techniques are unable to reach the exceedingly long timescales (i.e., minutes) that are relevant for protein biosynthesis. In this work, we present a novel coarse-grained approach that realistically models the ribosome/translocon/nascent-protein complex, while also allowing for the efficient simulation of biological timescales. The coarse-grained model is parameterized on the basis of extensive molecular dynamics simulations and enables the simulation of any nascent protein with only amino-acid sequence information as input. To validate the model, we perform over 26,000 simulations of protein biosynthesis, enabling direct comparison and demonstrating good agreement with a range of experimental studies describing this minute-timescale process.

## Introduction

Most integral membrane proteins (IMPs) are co-translationally inserted into the membrane during biosynthesis via the Sec translocon, a multiprotein complex [1–4]. In this process, a ribosome docks to the cytosolic opening of the Sec translocon and feeds a nascent polypeptide chain (NC) into the translocon channel. Secretory proteins, or the soluble domains of IMPs, translocate across the lipid membrane by passing through the translocon channel [1, 2]. Alternatively, the transmembrane domains (TMDs) of IMPs integrate directly into the lipid membrane via the translocon lateral gate (LG). Integration is facilitated by a conformational change in the channel that separates the two LG helices to create an opening between the channel interior and the hydrophobic core of the membrane [5–7]. The likelihood of integration or translocation of polypeptide segments depends on residue-specific chemical features of the nascent polypeptide chain, such as its hydrophobicity and charge [8–12], but is also governed by the dynamics of protein synthesis on the minute timescale [13, 14].

To reach a stable folded structure, IMPs must integrate into the membrane with the correct topology (i.e., orientation of each TMD with respect to the membrane), which depends sensitively on the properties of both the NC and the translocon itself [3, 15]. Even single mutations to an IMP amino-acid sequence can disrupt integration and induce disease phenotypes [16] or decrease protein expression [17–19]; similarly, mutations to the translocon channel can inhibit IMP folding [8, 20–23]. The important role for IMPs in cellular functions, such as signal transduction, the transport of nutrients, and cell adhesion, motivates the understanding of the effect of NC and translocon properties on the efficiency of co-translational integration. However, a detailed understanding of this process presents challenges for both theory and experiment due to the long range of timescales (from nanoseconds to minutes) that are involved.

Experimental studies have elucidated many aspects of the structure and function of the Sec translocon, although their ability to directly probe the non-equilibrium co-translational integration process is limited. Structural characterization has revealed many of the components of the translocon complex in both eukaryotes [24–28] and prokaryotes [6, 7, 29–32], while biophysical assays have investigated the functional effects of NC hydrophobicity [8, 9], charges flanking TMDs [10–12], soluble loop length [13, 14], and the forces exerted on a NC during translation [4, 33, 34]. Despite these findings, mechanistic details of the co-translational integration process remain in question [4] because most experiments are limited to probing final protein distributions—such as the fraction of protein in a specific topology [14] or the fraction of protein integrated in the membrane [35]—and do not typically resolve NC dynamics.

Atomistic-scale molecular dynamics simulations can be used to probe detailed aspects of co-translational integration, with recent simulations providing insight into the energetics of TMD integration [36, 37], the dynamics of water inside the translocon [38], the effect of NC properties on LG opening [39], the dynamics of a NC during the initial stages of translation [40–42], and the dynamics of IMP integration in simplified system representations [43, 44]. However, the separation of timescales relevant to co-translational integration poses a significant challenge to conventional simulation methods: notably, ribosomal translation requires seconds to minutes to complete the biosynthesis of typical polypeptides [45–48], while conformational fluctuations of the NC occur on the nanosecond timescale. Currently available simulation approaches either fail to reach the biological timescales of ribosomal translation [38, 40, 41] or lack sufficient detail to describe detailed features of the NC-translocon interactions and NC conformational dynamics [43, 44, 49]. The model presented here overcomes these limitations, allowing direct comparison with a broad range of available experiments.

In previous work, a highly coarse-grained (CG) model of Sec-facilitated IMP integration was developed in which all system coordinates are projected onto a two-dimensional plane passing through the translocon LG [43]. This 2D-CG model includes an explicit representation of NC translation, translocon LG conformational gating, and a sufficiently simple system description to enable minute-timescale unbiased trajectories. Previous work has demonstrated that the 2D-CG model correctly predicts the distribution of topologies obtained by TMDs as a function of C-terminal soluble loop length [43], the probability of membrane integration as a function of TMD hydrophobicity [43], the effect of charge mutations on the topology of the dual-topology protein EmrE [50], and the effect of sequence modifications on the integration efficiency of the multispanning protein TatC [19]. The 2D-CG model was also used to demonstrate a link between IMP integration efficiency and expression levels for TatC [19], enabling the computational prediction of amino-acid sequence modifications that improve IMP expression. These successes illustrate the potential for using CG methods to capture the essential physics of the co-translational protein translocation and membrane integration processes. However, several shortcomings of the 2D-CG model have been identified. In particular, the ribosome and translocon are modeled without detailed structural features, sequence-specific ribosome and translocon chemical features are not mapped directly to the CG representation, and interactions between the NC and the translocon are independent of NC sequence. These shortcomings limit the ability of the 2D-CG model to investigate phenomena arising from sequence-specific structural and chemical features, such as variations among homologs of the Sec translocon [6, 7] or interactions between the NC and translocon [51, 52].

In the current work, we describe a refined CG model that enables simulation of the long time- and length-scales that are relevant to co-translational protein integration, while preserving sequence-specific properties of the NC and translocon and capturing the structure of the ribosome-translocon complex. The new 3D-CG model extends the 2D-CG model by providing a realistic three-dimensional representation of the ribosome-translocon complex mapped directly from high-resolution structural data [6, 25]. Additionally, the model is parameterized via a bottom-up approach to reproduce sequence-specific NC-translocon interactions, and it includes a protocol for directly mapping any input amino-acid sequence to a simulation representation, enabling simulation of any polypeptide using only the amino-acid sequence as input. The improved 3D-CG model is validated by reproducing experimental measurements of TMD integration efficiency [51] and signal peptide topogenesis [14]. The model further reproduces the “biological hydrophobicity” scale derived by von Heijne and co-workers [51], capturing the effects of single-residue mutations on stop-transfer efficiency. The strong agreement between simulation and experiment indicates that the 3D-CG model produces simulation predictions that can be confirmed by direct experimental analogues. The new model provides a framework for performing mutagenesis studies of the NC and ribosome-translocon complex to obtain a detailed mechanistic understanding of interactions that impact TMD integration and topogenesis, potentially enabling the prediction of IMP sequence modifications with enhanced membrane integration efficiency and stability.

## Methods

We now present the details of the 3D-CG model of Sec-facilitated co-translational protein synthesis. The 3D-CG model preserves several features of the prior 2D-CG model [43], including (*i*) representation of the NC as a non-overlapping freely-jointed chain, (*ii*) 3:1 mapping of amino-acid residues to CG beads, (*iii*) implicit representation of the lipid membrane, (*iv*) stochastic opening and closing of the translocon LG, (*v*) explicit modeling of NC translation during the simulation trajectories, and (*vi*) sufficient computational efficiency to reach long second-minute timescales, achieved using a high level of coarse graining and the use of a partially tabulated potential energy function.

**Fig 1:**
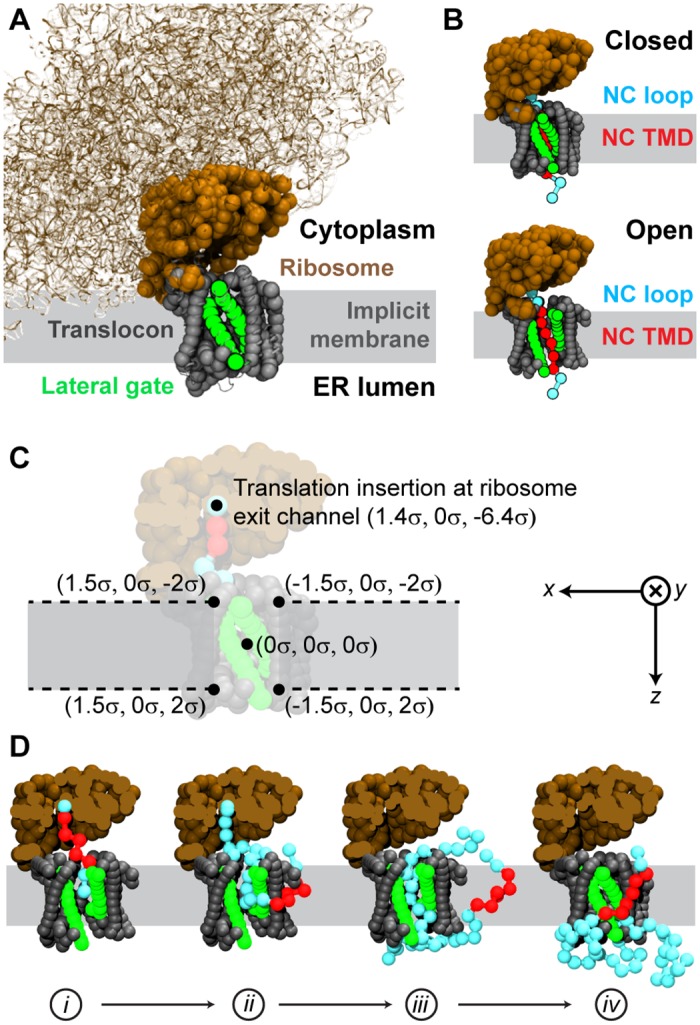
3D-CG model geometry. (A) Components of the 3D-CG model overlaid on a high-resolution cryo-EM structure of the ribosome-translocon complex [25]. 3D-CG model beads are represented by opaque spheres and are labeled according to their color. The region representing the implicit membrane is drawn as a grey background. (B) 3D-CG model snapshots of the two possible translocon conformations, with a closed lateral gate (top) and with an open lateral gate (bottom). In each case, a NC is shown emerging from the ribosome exit channel and interacting with the translocon. (C) Coordinate system for the 3D-CG model. Coordinates for the translation insertion point at the ribosome exit channel, the origin, and four points illustrating the bounds of the implicit membrane are indicated. (D) Simulation snapshots showing representative states during a simulation trajectory, including: (*i*) the start of translation, (*ii*) topological inversion of a TMD during integration, (*iii*) release of the C-terminus at the end of translation, and (*iv*) the end of a simulation in which the TMD has integrated into the membrane, the lateral gate is closed, and all polypeptide segments have exited the channel.

Significant improvements of the 3D-CG model described below include a three-dimensional representation of the ribosome/translocon/NC geometry (shown in Fig 1 and residue-specific interactions between the NC and the translocon. The resulting 3D-CG model allows any input amino-acid sequence to be directly converted to a CG simulation representation. The 3D-CG model then simulates the dynamics of the nascent protein, including elongation of the polypeptide during ribosomal translation, the integration of protein segments into the membrane bilayer, and the retention or translocation of protein segments flanking transmembrane domains (shown in Fig 1D).

### 3D-CG model geometry

Fig 1A presents the components of the 3D-CG model compared to an image of the ribosome-translocon complex obtained from a cryo-EM structure [25]. The SecYEG translocon (grey/green), ribosome (brown), and the NC (cyan/red) are represented with explicit CG beads, while the implicit membrane is drawn as a shaded region. As in the 2D-CG model [43], each CG bead has a diameter of *σ* = 0.8 nm, the Kuhn length of a polypeptide chain [43, 44], and represents three amino-acid residues; *σ* sets the length scale for the 3D-CG model. The coordinate system is defined such that the origin is placed at the geometric center of the translocon channel C*α* atoms, the implicit membrane spans the *x*-*y* plane with its midplane located at *z* = 0*σ*, and the axis of the translocon is aligned with the *z*-axis (Fig 1C).

The geometry of the Sec translocon is obtained by mapping all amino-acid residues of the translocon onto CG beads in a ratio of three amino acids to one CG bead, where the CG bead is positioned at the center of mass of the C*α* atoms for each consecutive triplet of amino-acid residues in the translocon primary sequence. Triplets of amino acids with a net positive charge are assigned a +1 charge, and triplets of amino acids with a net negative charge are assigned a -1 charge. To determine the net charge of a triplet of amino acids the charges of the amino acids are summed, with arginine and lysine counted as +1, and aspartate and glutamate counted as -1 (see S2 Appendix for further discussion). The translocon is modeled in two distinct conformations, with the LG either closed or open (Fig 1B). CG bead coordinates for both conformations are obtained from residue-based coarse-grained simulations of the *Methanocaldococcus jannaschii* SecYEG translocon (PDB ID: 1RHZ) [6] (see S2 Appendix). The 3D-CG model of the translocon is oriented such that the *y*-axis of the simulation coordinate system passes between the helices of the LG when the translocon is in the open conformation (Fig 1C).

The geometry of the ribosome is obtained by mapping the ribosome-translocon complex from a recent high-resolution cryo-EM structure (PDB ID: 3J7Q) onto CG beads [25]. Amino-acid residues are mapped onto CG beads in a 3:1 ratio following the same procedure used for the translocon. Each RNA nucleotide in the ribosome is mapped onto two CG beads; one bead represents the sugar-phosphate backbone, while the other bead represents the nucleobase. This mapping is used to capture the excluded volume and the rigidity of the RNA scaffold and is consistent with previous work on coarse-grained DNA/RNA simulations [53–55]. Each CG bead representing a RNA sugar-phosphate backbone in the ribosome is assigned a -1 charge and each CG bead representing a nucleobase is neutral. Only the portion of the ribosome near the translocon channel is explicitly represented as CG beads in the final simulation system (Fig 1A; additional details are in S2 Appendix). Ribosome CG bead positions are identical for both translocon conformations.

To characterize whether the *i*th NC bead, with position **x**_*i*_ = (*x*_*i*_, *y*_*i*_, *z*_*i*_), is located in the implicit membrane region, we define the characteristic function

$$S_{\text{mem}}\left(x_{i}\right)=\left[1-S\left(x_{i},y_{i}\right)\right]S\left(z_{i}\right),$$ (1)

which assumes a value of 1 in the membrane and 0 elsewhere. *S*(*x*, *y*) and *S*(*z*) are smooth switching functions,

$$S\left(x,y\right)=\frac{1}{4}\;\left[1+\text{tanh}\;\left(\frac{\sqrt{x^{2}+y^{2}}+1.5\sigma }{0.25\sigma }\right)\right]\;\left[1-\text{tanh}\;\left(\frac{\sqrt{x^{2}+y^{2}}-1.5\sigma }{0.25\sigma }\right)\right],$$ (2)

and

$$S\left(z\right)=\frac{1}{4}\;\left[1+\text{tanh}\;\left(\frac{z+2\sigma }{0.25\sigma }\right)\right]\;\left[1-\text{tanh}\;\left(\frac{z-2\sigma }{0.25\sigma }\right)\right],$$ (3)

where $\sqrt{x^{2}+y^{2}}$ is the radial distance from the coordinate system origin in the *x*-*y* plane. *S*(*x*, *y*) is approximately 1 for the range $-1.5\sigma <\sqrt{x^{2}+y^{2}}<1.5\sigma$ and 0 elsewhere, while *S*(*z*) is approximately 1 for the range -2*σ* < *z* < 2*σ* and 0 elsewhere (Fig 1C). Eqs 1–3 are used in Eq 8 to define the solvation of a NC bead.

### 3D-CG model potential energy function

The potential energy function for the 3D-CG model is expressed

$$U\left(x_{\text{n}},x_{\text{c}};q,g\right)=U_{\text{bond}}\left(x_{\text{n}}\right)+U_{\text{excl}}\left(x_{\text{n}}\right)+U_{\text{elec}}\left(x_{\text{n}},x_{\text{c}};q\right)+U_{\text{solv}}\left(x_{\text{n}};g\right)+U_{\text{chan}}\left(x_{\text{n}},x_{\text{c}};g\right)+U_{\text{ribo}}\left(x_{\text{n}}\right),$$ (4)

where **x**_n_ indicates the set of NC bead positions, **x**_c_ indicates the set of channel and ribosome bead positions, *q* is the set of all bead charges, and *g* is the set of all NC bead transfer free energies. All interactions in the 3D-CG model are defined using an energy scale given by *ϵ* = *k*_*B*_*T*, where the temperature, *T*, is fixed at 310 K to represent physiological conditions.

Bonded interactions between consecutive NC beads are described using the finite extension nonlinear elastic (FENE) potential,

$$U_{\text{bond}}\left(x_{n}\right)=-\frac{1}{2}K_{0}R_{0}^{2}\sum_{b\in \text{Bonds}}\text{ln}\;\left(1-\frac{r_{b}^{2}}{R_{0}^{2}}\right),$$ (5)

where the sum runs over all bonds in the NC, *r*_*b*_ is the distance between the NC beads that share bond *b*, *K*_0_ = 5.833 *ϵ*/*σ*^2^, and *R*_0_ = 2*σ*. Short-ranged excluded volume interactions between pairs of NC beads are modeled using a purely repulsive Lennard-Jones (LJ) potential [56],

$$U_{\text{excl}}\left(x_{n}\right)=\sum_{i,j\in \text{NC}}\;\left\{\begin{array}{ccc} 4\epsilon_{ij}\;\left[{\left(\frac{\sigma_{ij}}{r_{ij}}\right)}^{12}-{\left(\frac{\sigma_{ij}}{r_{ij}}\right)}^{6}\right]+\epsilon_{ij} & , & r_{ij}<2^{1/6}\sigma_{ij}\\ 0 & , & r_{ij}\geq 2^{1/6}\sigma_{ij} \end{array}\right\},$$ (6)

where the sum runs over all pairs of NC beads, *r*_*ij*_ is the distance between NC beads *i* and *j*, and *ϵ*_*ij*_ = *ϵ*, and *σ*_*ij*_ = *σ*.

Electrostatic interactions are described using the Debye-Hückel potential,

$$U_{\text{elec}}\left(x_{n},x_{c};q\right)=\sum_{i,j\in \text{All}}\;\frac{l_{B}q_{i}q_{j}\epsilon }{r_{ij}}\text{exp}\;\left(-\frac{r_{ij}}{\kappa }\right),$$ (7)

where the sum runs over all pairs of charged beads, *l*_*B*_ is the Bjerrum length, *q*_*i*_ is the charge of CG bead *i* in the NC, translocon, or ribosome, and *κ* is the Debye length. Assuming that electrostatic interactions are screened by physiological salt concentrations [57, 58], the electrostatic length scales are approximated by *κ* = *l*_*B*_ = *σ*.

NC bead interactions with the implicit solvent are described using a position-dependent potential,

$$U_{\text{solv}}\left(x_{n};g\right)=\sum_{i\in \text{NC}}g_{i}S_{\text{mem}}\left(x_{i}\right),$$ (8)

where **x**_*i*_ is the position of NC bead *i*, and *g*_*i*_ is the transfer free energy for partitioning NC bead *i* from water to the membrane.

Residue-specific interactions between NC beads and translocon beads are given by

$$U_{\text{chan}}\left(x_{n},x_{c};g\right)=\sum_{i\in \text{NC}}\left[1-S_{\text{mem}}\left(x_{i}\right)\right]U_{\text{chan}}^{\text{aq}}\left(x_{i},x_{c};g_{i}\right)+\left[S_{\text{mem}}\left(x_{i}\right)\right]U_{\text{chan}}^{\text{mem}}\left(x_{i},x_{c};g_{i}\right).$$ (9)

Eq 9 smoothly interpolates between NC bead-translocon interactions for which NC bead *i* is positioned in aqueous solution inside the channel ($U_{\text{chan}}^{\text{aq}}\left(x_{i},x_{c};g_{i}\right)$) or positioned in the membrane near the channel exterior ($U_{\text{chan}}^{\text{mem}}\left(x_{i},x_{c};g_{i}\right)$). The exact functional forms of $U_{\text{chan}}^{\text{aq}}\left(x_{i},x_{c};g_{i}\right)$ and $U_{\text{chan}}^{\text{mem}}\left(x_{i},x_{c};g_{i}\right)$ are described in the section *Parameterization of NC-translocon interactions*.

Interactions between NC beads and ribosome beads are included in the *U*_chan_(**x**_n_, **x**_c_; *g*) potential energy term (Eq 9). Contrary to interactions between NC beads and translocon beads, interactions between NC beads and ribosome beads are not bead-type specific; they are described by a repulsive soft-core LJ potential (Eq 17), with *ϵ*_*ij*_ = *ϵ* and *σ*_*j*_ = 1.2*σ*. To prevent the NC from moving into the part of the ribosome that is not explicitly included in the simulations (see *3D-CG Model Geometry*), a repulsive sphere is centered at (-10*σ*, -0.5*σ*, 1.0*σ*) (Fig 1C). Repulsive interactions with this sphere are described using

$$U_{\text{ribo}}\left(x_{n}\right)=\sum_{i\in \text{NC}}\;\left\{\begin{array}{ccc} 4\epsilon \;\left[{\left(\frac{\sigma }{r_{ir}-2\sigma }\right)}^{12}-{\left(\frac{\sigma }{r_{ir}-2\sigma }\right)}^{6}\right]+\epsilon & , & r_{ir}-2\sigma <2^{1/6}\sigma \\ 0 & , & r_{ir}-2\sigma \geq 2^{1/6}\sigma \end{array}\right\},$$ (10)

where *r*_*ir*_ is the distance of the NC bead *i* from the center of the sphere.

### 3D-CG model dynamics

The time evolution of the NC beads is modeled using overdamped Langevin dynamics with a first-order Euler integrator [59],

$$x_{n}\left(t+\Delta t\right)=x_{n}\left(t\right)-\beta D\nabla_{x_{n}}U\left(x_{n}\left(t\right),x_{c}\left(t\right);q,g\right)\Delta t+\sqrt{2D\Delta t}R\left(t\right),$$ (11)

where **x**_n_(*t*) are the positions of the NC beads at time *t*, *U*(**x**_n_(*t*), **x**_c_(*t*); *q*, *g*) is the 3D-CG model potential energy function (Eq 4), *β* = 1/*k*_*B*_*T*, *D* is an isotropic diffusion coefficient, and **R**(*t*) is a random number vector drawn from a Gaussian distribution with zero mean and unit variance. The timestep, Δ*t* = 300 ns, permits stable integration of the equations of motion with a diffusion coefficient of *D* = 253.0 nm^2^/s (see S3 Appendix for discussion and Table S2 in S3 Appendix for robustness with respect to timestep). Ribosome CG bead coordinates are fixed throughout the simulations. Translocon CG beads undergo stochastic transitions between fixed configurations associated with the open versus closed lateral gate.

NC-dependent conformational gating of the translocon is attempted at every simulation timestep. The probability that the translocon transitions from a closed ($x_{c}^{\text{closed}}$) to open ($x_{c}^{\text{open}}$) conformation, *p*_open_(**x**_n_; *q*, *g*), is

$$p_{\text{open}}\left(x_{n};q,g\right)=\frac{1}{\tau_{\text{LG}}}\frac{\text{exp}\;\left[-\beta \Delta G_{\text{open}}\left(x_{n};q,g\right)\right]}{1+\text{exp}\;\left[-\beta \Delta G_{\text{open}}\left(x_{n};q,g\right)\right]}\Delta t,$$ (12)

and the probability that the translocon transitions from an open to closed conformation, *p*_close_(**x**_n_; *q*, *g*), is

$$p_{\text{close}}\left(x_{n};q,g\right)=\frac{1}{\tau_{\text{LG}}}\frac{1}{1+\text{exp}\;\left[-\beta \Delta G_{\text{open}}\left(x_{n};q,g\right)\right]}\Delta t.$$ (13)

The timescale for attempting translocon conformational changes, *τ*_LG_ = 500 ns, is obtained from prior molecular dynamics simulations [39, 43]. The total free energy change for switching the translocon from the closed to open conformation, Δ*G*_open_(**x**_n_; *q*, *g*), is given by

$$\Delta G_{\text{open}}\left(x_{n};q,g\right)=\Delta G_{\text{empty}}+U\left(x_{n},x_{c}^{\text{open}};q,g\right)-U\left(x_{n},x_{c}^{\text{closed}};q,g\right),$$ (14)

where Δ*G*_empty_ = 3*ϵ* is the free energy penalty for opening a closed channel in the absence of a substrate [60], $U(x_{n},x_{c}^{\text{open}};q,g)$ is the 3D-CG model potential energy function (Eq 4) with the channel in the open configuration, and $U(x_{n},x_{c}^{\text{closed}};q,g)$ is the 3D-CG model potential energy function (Eq 4) with the channel in the closed configuration. Previous simulations have found the translocon to exhibit both closed and open lateral-gate conformations [39], and the timescale needed to perform this conformational switch is relatively small (500 ns) in comparison to the other timescales modeled in the 3D-CG model [40]. Therefore, as in the 2D-CG model [43], the lateral-gate conformational changes in the 3D-CG model are described in terms of instantaneous switches between the closed and open conformations. If an attempted conformational change is accepted, all bead positions in the translocon are immediately switched to the positions corresponding to the new channel conformation. The equations of motion described by Eqs 11–14 rigorously obey detailed balance.

Translation of the NC is modeled by adding CG beads to the C-terminus of the NC during a simulation trajectory. At the initiation of the trajectory, the C-terminal NC bead is fixed at the translation insertion point (Fig 1C). For each simulation timestep in which translation is performed, the C-terminal bead is moved in the +*z* direction by a distance equal to *σ*Δ*t*/*t*_trans_, where *t*_trans_ is the timescale for translating a single CG bead. *t*_trans_ is set to 0.6 seconds to reproduce a translation rate of 5 residues/second [45–48] unless otherwise specified. The C-terminal NC bead is otherwise held fixed, although all interactions between the C-terminal NC bead and other NC beads are included in Eq 4. The translation of the C-terminal bead is completed after a period of *t*_trans_ and its dynamics are described using Eq 11 for the remainder of the simulation trajectory. The next CG bead in the NC sequence is then positioned at the translation insertion point and the process is repeated until all NC beads have been translated.

For the combined dynamics of the ribosome-translocon-NC system, a series of five steps is iterated at each trajectory timestep: (*i*) forces acting on each NC bead are calculated, (*ii*) NC bead positions are time-evolved using Eq 11, (*iii*) conformational gating of the translocon is attempted (Eqs 12 and 13), (*iv*) ribosomal translation is performed if all NC beads have not yet been translated, and (*v*) the simulation is terminated if user-defined conditions are met. Specific protocols for initializing and terminating simulation trajectories are provided for each workflow described in the *Results*.

### 3D-CG model parameterization

While the system geometry, 3D-CG model dynamics, and most terms in the 3D-CG model potential energy function (Eq 4) are fully described in the *Methods*, the functional forms of the NC-translocon interaction potentials, $U_{\text{chan}}^{\text{aq}}\left(x_{i},x_{c};g_{i}\right)$ and $U_{\text{chan}}^{\text{mem}}\left(x_{i},x_{c};g_{i}\right)$ in Eq 9, have yet to be specified. Here, we describe the protocol for obtaining these potentials, which determine sequence-specific NC bead-translocon interactions.

First, we define a protocol for assigning an effective water-membrane transfer free energy, *g*_*i*_, and charge, *q*_*i*_, to a NC bead, based on available experimental data. Second, potentials of mean force (PMFs) for translocating model tripeptide substrates across the translocon channel are calculated using the MARTINI residue-based coarse-grained force field. Finally, sequence-specific NC bead-translocon interactions in the 3D-CG model are parameterized by reproducing the MARTINI PMFs using the 3D-CG potential energy function.

#### Determination of substrate water-membrane transfer free energies and charge

The water-membrane transfer free energy, or hydrophobicity, of a NC bead, *g*_*i*_, is calculated by summing the transfer free energies of the associated trio of amino-acid residues. Residue-specific transfer free energies are obtained from the Wimley-White octanol-water hydrophobicity scale, which measures the partitioning of pentapeptides between octanol and water in a well-defined experimental assay [61]. The Wimley-White hydrophobicity scale has been shown to correlate well with other biophysical hydrophobicity scales [51, 62, 63]. Hydrophobic residues have negative transfer free energies while hydrophilic and charged residues have positive transfer free energies; the full hydrophobicity scale is reproduced in units of *ϵ* in Table S1 of S3 Appendix. The Wimley-White hydrophobicity scale assumes that each residue’s peptide backbone participates in intramolecular hydrogen bonds typical of residues forming secondary structure elements. Peptide bonds that do not form intramolecular hydrogen bonds have an additional free energy cost for partitioning into the membrane [61, 64, 65]. Hence, the transfer free energy of a residue is increased by 1.78*ϵ*, the approximate cost for partitioning a peptide bond that lacks hydrogen bonds, if it is assumed to not form a secondary structure element as discussed in the section *Mapping amino-acid sequence properties to CG beads*.

The charge of a NC bead, *q*_*i*_, is equal to the sum of the charges of the three associated amino-acid residues. It is assumed that arginine and lysine residues bear a +1 charge, glutamate and aspartate residues bear a -1 charge, and all other residues are neutral. The N- and C-terminal CG beads are assigned an additional +1 and -1 charge, respectively, and have 6*ϵ* added to their transfer free energies to account for the additional charge [66].

#### Residue-based coarse-grained simulations

**Fig 2:**
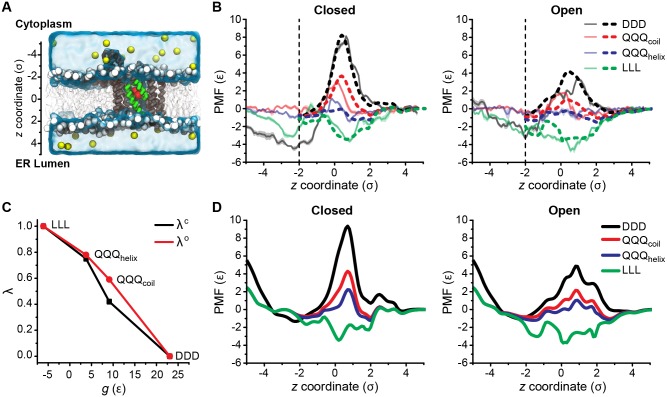
Bottom-up parameterization of NC bead-translocon interactions. (A) Simulation snapshot of the residue-based coarse-grained simulation system using the MARTINI force field. The translocon is in its closed conformation, a tripeptide substrate is shown in red, lipids are shown with head groups in white and tail groups in grey, water is represented as a transparent surface, and ions are shown as yellow spheres. (B) PMFs for translocating homogeneous tripeptides across the closed (left) and open (right) channel conformations. PMFs calculated using MARTINI for all four tripeptides are plotted as transparent lines, with shaded regions indicating the estimated error. The MARTINI PMFs are scaled by a factor of 0.25 and are vertically shifted such that the average value for 4.0*σ* ≤ *z* ≤ 4.5*σ* is 0. Best-fit PMFs calculated using the 3D-CG model are plotted as opaque dashed lines, and are fit in the range *z* ≥ −2*σ* (dashed vertical line). All PMFs are presented as a function of *z*, rather than *d*_*z*_, since these values differ only by an offset of 0.1*σ*. (C) Piecewise linear interpolation relating values of *λ*^c^ and *λ*^o^ to the substrate hydrophobicity *g*. The endpoints of the piecewise linear interpolation correspond to the four substrates in B. (D) PMFs calculated using the 3D-CG model and the best-fit parameters, for the same four peptides as in B, but with the ribosome and translocon plug domain included.

Residue-based coarse-grained simulations are performed using the MARTINI force field, version v2.2P, with the MARTINI polarizable water model [67, 68]. In the MARTINI model, each amino-acid residue is represented by a backbone particle and one or more side-chain particles. MARTINI simulations include the translocon embedded within a lipid membrane containing 368 palmitoyloleoylphosphatidylcholine (POPC) lipids and solvated by an electroneutral 50 mM NaCl salt solution containing 6,225 CG polarizable water molecules (Fig 2A). The ribosome is not included due to its large size, and the plug region (Ala^48^-Leu^70^) was excluded from the MARTINI representation of the continuous translocon sequence to avoid slow-timescale sampling issues [39]. The translocon is restrained during these simulations to either the closed or open conformation by applying a biasing potential; the minimum distance between any pair of backbone particles in separate LG helices is restrained to be 0.88*σ* in the closed conformation and 1.75*σ* in the open conformation based on previous molecular dynamics results [39]. The described simulation system is used to determine bead positions for the 3D-CG model channel geometry (*3D-CG Model Geometry*) and for PMF calculations. Complete details on the MARTINI simulations, collective variable definitions, and PMF calculation are provided in S1 Appendix.

PMFs for translocating homogeneous tripeptide substrates through the translocon are calculated from umbrella-sampling simulations. The collective variable, *d*_*z*_, is defined as the distance along the *z*-axis (i.e., the channel axis) between the center-of-mass of the tripeptide and the center-of-mass of the six hydrophobic pore residues in the translocon [I75, V79, I170, I174, I260, L406] (Fig 2A and Fig S1 of S1 Appendix). In each umbrella-sampling trajectory, the substrate is kept near a specific value of *d*_*z*_ using a harmonic restraint, confined within a cylinder of radius 1.5*σ*, and sampled for 400 ns. At least 50 umbrella-sampling trajectories, spanning a range of *d*_*z*_ values between −5.0*σ* and 4.5*σ*, are performed for each substrate. Additional simulation trajectories are generated for a restricted range of *d*_*z*_ to improve convergence as needed (summarized in Table S1 of S1 Appendix). Each translocation PMF is obtained from the set of corresponding umbrella-sampling trajectories using the Weighted Histogram Analysis Method [69]. Additional details on the umbrella-sampling simulations are provided in S1 Appendix section *MARTINI simulations for translocation PMF profiles*.

Translocation PMFs are calculated for homogeneous leucine (LLL), glutamine (QQQ), and aspartate (DDD) tripeptides. These substrates are selected because their water-membrane transfer free energies span a range from very hydrophobic (LLL) to very hydrophilic (DDD). In the MARTINI force field, each residue is represented by a backbone particle and one or more side chain particles, with the backbone particle type assigned based on the secondary structure of the residue. The LLL substrate is assigned the more hydrophobic “helix” backbone type, the DDD substrate is assigned the more hydrophilic “coil” backbone type, and the QQQ substrate, of intermediate hydrophobicity, is simulated twice, once with the helix backbone type (QQQ_helix_) and once with the coil backbone type (QQQ_coil_). The difference in backbone particle type affects only the non-bonded interactions between the backbone particle and other particles; given the short length of the tripeptides, the change in the backbone type does not affect tripeptide structure.

Fig 2B shows PMFs calculated from the MARTINI simulations for the translocation of all four substrates and both channel conformations. Previous work has shown that amino-acid water-lipid transfer free energies calculated using MARTINI correlate well with the Wimley-White transfer free energy scale, but the correlation has a slope of 3.69 [63]; to treat NC-lipid interactions and NC-translocon interactions in the 3D-CG model on an equal footing, the MARTINI PMFs are rescaled by a factor of 0.25 and the rescaled PMFs are presented in Fig 2B. The hydrophobic LLL substrate (green in Fig 2) and hydrophilic DDD substrate (black in Fig 2) demonstrate opposing behavior in both channel conformations; LLL is attracted to the center of the channel, which is lined with hydrophobic residues [6, 8], while DDD is repelled. These results qualitatively agree with the atomistic simulations of similar substrates performed by Gumbart et al. [36, 70]. The more hydrophobic QQQ_helix_ substrate is more attracted to the center of the channel than the QQQ_coil_ substrate, while PMFs for both QQQ substrates lie in between the LLL and DDD PMFs. These results show that NC bead-channel interaction ranges from attractive to repulsive as the substrate becomes more hydrophilic.

#### Parameterization of NC-translocon interactions

Residue-specific NC bead-translocon interactions (Eq 9) are obtained by parameterizing the 3D-CG model to fit the MARTINI PMFs shown in Fig 2B. Based on the MARTINI results, we assume that: (*i*) NC bead-translocon interactions are a function of substrate hydrophobicity, (*ii*) interactions with the LLL and DDD tripeptides represent the most attractive and most repulsive possible channel interactions, respectively, and (*iii*) all other NC bead-translocon interactions vary between these extremes. Further, we assume that $U_{\text{chan}}^{\text{mem}}\left(x_{i},x_{c};g_{i}\right)$ is independent of NC bead properties. Therefore, the $U_{\text{chan}}^{\text{aq}}\left(x_{i},x_{c};g_{i}\right)$ term in Eq 9, which describes pairwise interactions between NC bead *i* and channel bead *j*, is decomposed into four separate interactions, given by

$$U_{\text{chan}}^{\text{aq}}\left(x_{i},x_{c};g_{i}\right)=\sum_{j\notin \text{NC}}\;\left\{\begin{array}{ccc} \lambda^{o}\left(g_{i}\right)U_{\text{LLL}}^{\text{open}}\left(r_{ij}\right)+\left[1-\lambda^{o}\left(g_{i}\right)\right]U_{\text{DDD}}^{\text{open}}\left(r_{ij}\right) & , & \text{open}\;\text{channel}\\ \lambda^{c}\left(g_{i}\right)U_{\text{LLL}}^{\text{closed}}\left(r_{ij}\right)+\left[1-\lambda^{c}\left(g_{i}\right)\right]U_{\text{DDD}}^{\text{closed}}\left(r_{ij}\right) & , & \text{closed}\;\text{channel} \end{array}\right\},$$ (15)

and the $U_{\text{chan}}^{\text{mem}}\left(x_{i},x_{c};g_{i}\right)$ term in Eq 9 contains a single term that is not bead-type dependent

$$U_{\text{chan}}^{\text{mem}}\left(x_{i},x_{c};g_{i}\right)=\sum_{j\notin \text{NC}}U_{\text{out}}\left(r_{ij}\right),$$ (16)

where *r*_*ij*_ is the distance between NC bead *i* and translocon channel bead *j*, $U_{\text{LLL}}^{\text{open}}\left(r_{ij}\right)$ and $U_{\text{LLL}}^{\text{closed}}\left(r_{ij}\right)$ are the interactions in the 3D-CG model between a NC bead representing a LLL tripeptide and the open or closed channel, respectively, and $U_{\text{DDD}}^{\text{open}}\left(r_{ij}\right)$ and $U_{\text{DDD}}^{\text{closed}}\left(r_{ij}\right)$ are the interactions in the 3D-CG model between a NC bead representing a DDD tripeptide and the open or closed channel, respectively. *λ*^o^(*g*_*i*_) and *λ*^c^(*g*_*i*_) are NC bead-specific parameters that interpolate the channel interactions for NC bead *i* between the most attractive interaction ($U_{\text{LLL}}^{\text{open}}\left(r_{ij}\right)$ for *λ*^o^(*g*_*i*_) = 1 or $U_{\text{LLL}}^{\text{closed}}\left(r_{ij}\right)$ for *λ*^c^(*g*_*i*_) = 1) to the most repulsive interaction ($U_{\text{DDD}}^{\text{open}}\left(r_{ij}\right)$ for *λ*^o^(*g*_*i*_) = 0 or $U_{\text{DDD}}^{\text{closed}}\left(r_{ij}\right)$ for *λ*^c^(*g*_*i*_) = 0), depending on the bead hydrophobicity, *g*_*i*_.

The functional form for *U*_out_(*r*_*ij*_), $U_{\text{LLL}}^{\text{open}}\left(r_{ij}\right)$, $U_{\text{LLL}}^{\text{closed}}\left(r_{ij}\right)$, $U_{\text{DDD}}^{\text{open}}\left(r_{ij}\right)$, and $U_{\text{DDD}}^{\text{closed}}\left(r_{ij}\right)$ is a soft-core LJ potential with three free parameters per unique channel bead [71],

$$U\left(r_{ij}\right)=\left\{\begin{array}{ccc} 4\epsilon_{j}^{\text{int}}\;\left(\frac{1}{{\left[\alpha_{j}+{\left(r_{ij}/\sigma_{j}\right)}^{6}\right]}^{2}}-\frac{1}{\left[\alpha_{j}+{\left(r_{ij}/\sigma_{j}\right)}^{6}\right]}\right)-\epsilon_{j}^{\text{cr}} & , & r_{ij}<r_{ij}^{\text{cr}}\\ 0 & , & r_{ij}\geq r_{ij}^{\text{cr}} \end{array}\right\},$$ (17)

where $\epsilon_{j}^{\text{int}}$ is the interaction energy, $r_{ij}^{\text{cr}}$ is the right cut-off radius, *σ*_*j*_ is the diameter of channel bead *j*. The term $\epsilon_{j}^{\text{cr}}$ is the value of the potential at the right cut-off radius, and $\alpha_{j}=0.02\left(\epsilon_{j}^{\text{int}}/\epsilon \right)\;[\sqrt{1+100(\epsilon /\epsilon_{j}^{\text{int}})}-1]$ is chosen to cap the maximum value of the potential to prevent infinite energies during the stochastic gating of the translocon conformation, as described in *3D-CG Model Dynamics* (Eq 14). For the beadtype independent interactions with the channel exterior, *U*_out_(*r*_*ij*_), we assign the free parameters $\epsilon_{j}^{\text{int}}$, $r_{ij}^{\text{cr}}$, and *σ*_*j*_ to represent interactions between NC beads and the hydrophobic channel exterior in a lipid environment (Table 1). For the beadtype dependent interactions with the channel interior, $U_{\text{chan}}^{\text{aq}}\left(x_{i},x_{c};g_{i}\right)$, the free parameters, $\epsilon_{j}^{\text{int}}$, $r_{ij}^{\text{cr}}$, and *σ*_*j*_ are fit for each of the four potential energy terms in Eq 15, as described below.

**Table 1:** Parameters defining NC-translocon interactions.

Potential	$\epsilon_{j}^{\text{int}}\left[\epsilon \right]$	$\epsilon_{j}^{\text{cr}}\left[\epsilon \right]$	$r_{j}^{\text{cr}}\left[\sigma \right]$	*α*_*j*_[*σ*]	*σ*_*j*_[*σ*]
$U_{\text{LLL}}^{\text{open}}$	0.46	-0.008	2.500	0.127	1.0
$U_{\text{LLL}}^{\text{closed}}$	0.30	-0.005	2.500	0.104	1.0
$U_{\text{DDD}}^{\text{open}}$	0.30	-0.005	2.500	0.104	1.0
$U_{\text{DDD}}^{\text{closed}}$	0.30	-0.005	2.500	0.104	1.0
$U_{\text{LLL}}^{\text{open}-\text{confined}}$	1.38	-0.023	2.500	0.209	1.0
$U_{\text{LLL}}^{\text{closed}-\text{confined}}$	1.41	-0.023	2.500	0.211	1.0
$U_{\text{DDD}}^{\text{open}-\text{confined}}$	9.85	-9.85	1.075	0.461	1.2
$U_{\text{DDD}}^{\text{closed}-\text{confined}}$	0.51	-0.51	1.110	0.133	1.2
*U*_out_	0.50	-0.008	2.500	0.132	1.0

In order to find parameters for the 3D-CG model that best reproduce the MARTINI PMF data, corresponding PMFs must be calculated using the 3D-CG model. The PMF for translocating a single CG bead, *i*, across the channel in the 3D-CG model can be calculated using numerical integration if all interactions for that NC bead with the channel and solvent are defined. As all potential terms other than $U_{\text{chan}}^{\text{aq}}\left(x_{i},x_{c};g_{i}\right)$ (Eq 15) are now defined, the MARTINI PMF data is used to define the remaining potential terms. First, parameters for $U_{\text{LLL}}^{\text{open}}\left(r_{ij}\right)$, $U_{\text{LLL}}^{\text{closed}}\left(r_{ij}\right)$, $U_{\text{DDD}}^{\text{open}}\left(r_{ij}\right)$, and $U_{\text{DDD}}^{\text{closed}}\left(r_{ij}\right)$, are determined independently by fixing the channel in a single conformation, either open or closed, and setting the value of *λ*(*g*_*i*_) to either 1 or 0 such that only one of the potential terms contributes to the interactions with CG bead *i*. Specifically, for the open channel configuration, a PMF calculated with *λ*^o^(*g*_LLL_) = 1, where *g*_LLL_ = -6.1*ϵ* is the water-lipid transfer free energy for a LLL substrate, is fit to the MARTINI PMF for LLL in the open channel to determine parameters for $U_{\text{LLL}}^{\text{open}}\left(r_{ij}\right)$. A PMF calculated with *λ*^o^(*g*_DDD_) = 0, where *g*_DDD_ = 23.1*ϵ* is the water-lipid transfer free energy for a DDD substrate, is fit to the MARTINI PMF for DDD in the open channel to determine parameters for $U_{\text{DDD}}^{\text{open}}\left(r_{ij}\right)$. Similarly, for the closed channel configuration, a PMF calculated with *λ*^c^(*g*_LLL_) = 1 is fit to the MARTINI PMF for LLL in the closed channel to determine parameters for $U_{\text{LLL}}^{\text{closed}}\left(r_{ij}\right)$, and a PMF calculated with *λ*^c^(*g*_DDD_) = 0 is fit to the MARTINI PMF for DDD in the closed channel to determine parameters for $U_{\text{DDD}}^{\text{closed}}\left(r_{ij}\right)$. We find that fitting the MARTINI PMFs requires at least two bead types for the translocon channel; one “normal” bead type, and one “confined” bead type, that have distinct parameter values. The values for all resulting parameters are summarized in Table 1. Details for the fitting process and the assignment of channel bead types are included in S3 Appendix. Fig 2B shows the best-fit PMFs calculated using numerical integration for the 3D-CG model potential energy function with the parameters listed in Table 1 (opaque dashed lines) overlaid on the corresponding MARTINI PMFs (transparent solid lines).

Having obtained parameters for *U*_out_(*r*_*ij*_), $U_{\text{LLL}}^{\text{open}}\left(r_{ij}\right)$, $U_{\text{LLL}}^{\text{closed}}\left(r_{ij}\right)$, $U_{\text{DDD}}^{\text{open}}\left(r_{ij}\right)$, and $U_{\text{DDD}}^{\text{closed}}\left(r_{ij}\right)$, we define a mapping between the transfer free energy (*g*_*i*_) of any NC bead and its corresponding channel interactions (*λ*^o^(*g*_*i*_) and *λ*^c^(*g*_*i*_)) to fully specify Eq 15. These mappings for the LLL, DDD, QQQ_helix_, and QQQ_coil_ substrates are determined by fitting the MARTINI PMFs. For a CG bead with an arbitrary value of *g*_*i*_, the corresponding value of *λ*^o^(*g*_*i*_) and *λ*^c^(*g*_*i*_) is determined by linear interpolation between these four points. As described previously, the values of *λ*^o^(*g*_*i*_) and *λ*^c^(*g*_*i*_) for the LLL substrate are set to 1, the values of *λ*^o^(*g*_*i*_) and *λ*^c^(*g*_*i*_) for the DDD substrate are set to 0. For QQQ_helix_ and QQQ_coil_ the values of *λ*^o^(*g*_*i*_) and *λ*^c^(*g*_*i*_) are determined as follows. First, the channel is fixed in the open conformation and the PMF for translocating a QQQ_helix_ substrate across the open channel in the 3D-CG model is calculated using numerical integration. The QQQ_helix_ 3D-CG model PMF is then fit to the MARTINI PMF for translocating the QQQ_helix_ substrate across the open channel, with *λ*^o^(*g*_QQQ_) as a fitting parameter, where *g*_QQQ_ = 3.8*ϵ* is the water-lipid TFE of a QQQ helix bead. This procedure is repeated for translocating a QQQ_helix_ substrate across the closed channel to obtain a best-fit value of *λ*^c^(*g*_QQQ_) for the QQQ_helix_ substrate.

Next, the transfer free energy for the QQQ_coil_ CG bead in the 3D-CG model is assigned by increasing the transfer free energy for the QQQ_helix_ CG bead by 5.3*ϵ*, which is the cost for partitioning three peptide bonds that lack hydrogen bonds between water and alkane (see *Determination of Substrate Water-Membrane Transfer Free Energies*) [61, 64, 65]. PMFs for translocating the QQQ_coil_ substrate across both the open and closed channels for the 3D-CG model are calculated using numerical integration and fit to the corresponding MARTINI PMFs to obtain best-fit values of *λ*^o^(*g*_QQQc_) and *λ*^c^(*g*_QQQc_), where *g*_QQQc_ = 9.1*ϵ* is the water-lipid transfer free energy of a QQQ_coil_ bead. Best-fit values of the translocation PMFs for the QQQ_helix_ and QQQ_coil_ substrates are shown in Fig 2B.

Having obtained *λ*^o^(*g*_*i*_), and *λ*^c^(*g*_*i*_) values for LLL, DDD, QQQ_coil_, and QQQ_helix_ by direct fitting to the MARTINI PMF profiles, a piecewise linear interpolation between these four sets of *g*_*i*_, *λ*^o^(*g*_*i*_), and *λ*^c^(*g*_*i*_) values is then performed to define values of *λ*^o^(*g*_*i*_), and *λ*^c^(*g*_*i*_) for a CG bead with an arbitrary value of *g*_*i*_, as shown in Fig 2C. In principle, this mapping between CG bead hydrophobicity and channel interactions could be further refined by simulating translocation PMFs with the MARTINI force field for all possible tripeptide substrates, including heterogeneous tripeptides, and then fitting independent channel interactions in the 3D-CG model for each tripeptide; however, due to the significant computational expense of the MARTINI calculations, we use the piecewise linear interpolation scheme specified above, which yields good agreement with experiments (see Results). Future work may further refine the relationship between substrate properties and channel interactions.

The bottom-up parameterization process completely specifies all terms in the 3D-CG potential energy function that define interactions between a CG bead with hydrophobicity *g*_*i*_ and the translocon channel. One caveat is that all translocation PMFs used in the fitting procedure are calculated in the absence of the ribosome and plug domain, which are present in the full 3D-CG model. Fig 2D shows PMFs calculated using numerical integration for the same four tripeptide substrates using the 3D-CG model with best-fit values, and including the ribosome and plug domain. Comparing Fig 2B and 2D shows that the plug domain does not have a large effect on the PMF. The only minor effect associated with including the plug domain appears to be a small shift in the position of the barrier for QQQ_helix_ with the translocon in the closed configuration; inclusion of the ribosome has no observable effect on the PMFs. The final PMFs, presented in Fig 2D, are thus representative of the interactions of CG beads with the translocon during 3D-CG model simulations.

### Mapping amino-acid sequence properties to CG beads

**Fig 3:**
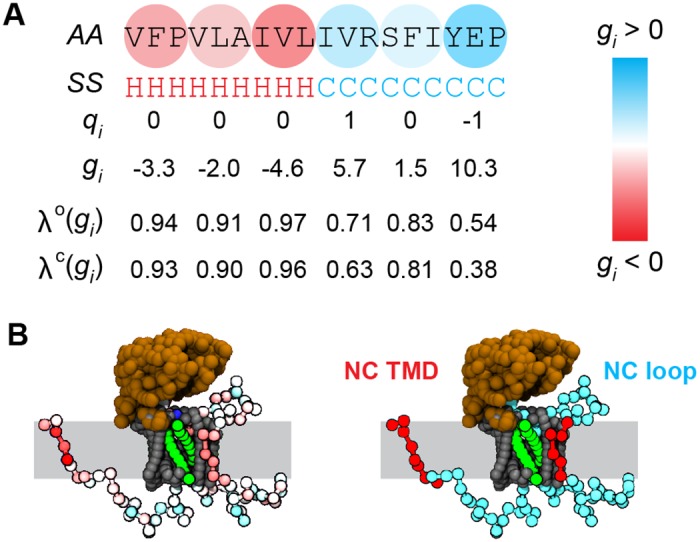
Example sequence mapped to 3D-CG model representation. (A) An input amino-acid sequence (*AA*) and secondary structure assignments (*SS*; H for helix and C for coil) are mapped to 3D-CG beads and assigned values of *q*_*i*_, *g*_*i*_, *λ*^c^(*g*_*i*_), and *λ*^o^(*g*_*i*_) based on the properties of sequential amino-acid triplets. (B) Visualization of heterogeneous NC properties and correspondence with structural elements. Left, a snapshot of a NC with each CG bead colored by *g*_*i*_; red beads are hydrophobic, while cyan beads are hydrophilic. Right, the same snapshot colored by assigning each NC bead to a domain.

The interactions between a general NC bead and the rest of the system is defined by four parameters: *g*_*i*_, *q*_*i*_, *λ*^o^(*g*_*i*_), and $\lambda_{i}^{c}\left(g_{i}\right)$. These parameters are determined as described in detail in section *3D-CG Model Parameterization*. Specifically, the NC bead transfer free energy, *g*_*i*_, is equal to the sum of the transfer free energies of the three amino-acid residues associated with the bead according to the Wimley-White hydrophobicity scale (Table S1 in S3 Appendix). For each residue that does not form secondary structure, *g*_*i*_ is increased by 1.78*ϵ*, the cost for partitioning a peptide bond that lacks hydrogen bonds. The CG bead charge, *q*_*i*_, is equal to the sum of the charges of the three associated amino-acid residues. The N- and C-terminal CG beads are assigned an additional +1 and -1 charge, respectively, and have 6*ϵ* added to their transfer free energies to account for the additional charge [66]. The scaling parameters for NC-channel interactions, *λ*^o^(*g*_*i*_) and *λ*^c^(*g*_*i*_), are determined from *g*_*i*_ using the piecewise-linear interpolation scheme shown in Fig 2C. Fig 3 demonstrates the mapping procedure for an example amino-acid sequence.

To start a 3D-CG simulation, both an input amino acid-sequence and a secondary structure assignment for this sequence must be provided. For the membrane integration simulations, the secondary structure of the experimental sequence is reported in the UniProt database and is assigned in the model directly from the available information [72]. For simulations of TMD topology, the secondary structure is not available through the UniProt database and is instead assigned using the PSIPRED secondary structure prediction server [73].

## Results and discussion

Having fully specified the features and parameters of the 3D-CG model, we now validate the model by simulating three biophysical assays and comparing the simulation results to previously published experimental data. The CG model is used to calculate (*i*) the probability of membrane integration as a function of NC segment hydrophobicity [51], (*ii*) the residue-specific change in the probability of membrane integration (i.e., the “biological hydrophobicity scale”) for all twenty amino-acid residues [51], and (*iii*) the distribution of final topologies of a hydrophobic TMD as a function of C-terminal soluble loop length and translation rate [14]. Together, these tests demonstrate the ability of the 3D-CG model to correctly predict the integration and orientation of TMDs with minimal input, as well as the effect of sequence mutations.

### Probability of membrane integration for NC segments of varying hydrophobicity

TMDs typically contain a large number of hydrophobic residues to improve stability within the lipid membrane [74]. von Heijne and co-workers measured the probability with which a designed segment (H-segment) of the leader peptidase (Lep) protein integrates into the membrane, demonstrating that the translocon is more likely to integrate hydrophobic NC segments [51]. It was found that increasing the hydrophobicity of a poly-alanine H-segment, through mutation of alanine residues to leucine residues, monotonically increased the probability of H-segment membrane integration. Previous simulations using model sequences and the 2D-CG simulation model revealed that this trend is caused by local equilibration of the H-segment across the translocon lateral gate [43]. Reproducing the same assay using the 3D-CG model, with full structural detail and an direct mapping of the NC amino acid sequence, provides a first means to quantitatively validate model predictions.

**Fig 4:**
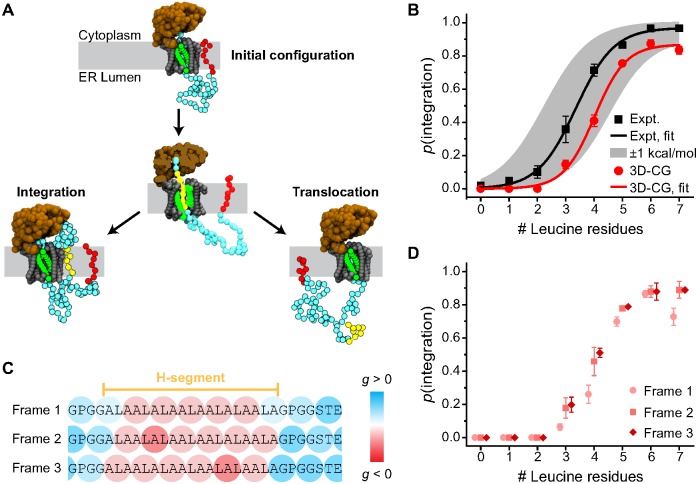
3D-CG model predictions of membrane integration versus secretion. (A) Snapshots of the initial system configuration, an intermediate state in which the H-segment (yellow) enters the channel, and two possible simulation products. Simulations are initialized with the TMD upstream of the H-segment (red) integrated into the membrane. (B) Probability of membrane integration (*p*(integration)) as a function of the number of leucine residues in the H-segment. Experimental results from Hessa et al. [51] are reproduced in black, while results from the 3D-CG model are shown in red. Each point for the 3D-CG model is the average of all three frameshifts. The solid lines are sigmoidal fits to each data set. (C) Schematic representation of three possible 3D-CG representations of the same sequence (i.e., frameshifts). The example sequence is the Lep construct with a 7 leucine H-segment (identified in yellow region). Each triplet is colored according to its value of *g*. (D) Probability of membrane integration as a function of the number of leucine residues in the H-segment for each individual frameshift.

To simulate the H-segment membrane integration assay with the 3D-CG model, the Lep protein sequence is mapped to CG beads following the procedure described in *Mapping amino-acid sequence properties to CG beads*. Three helical secondary structure elements, including the H-segment are identified via the UniProt database (ID:P00803). Eight 19-residue H-segments are studied. Each H-segment contains between 0 to 7 leucine residues and the remaining H-segment residues are alanine [51]. All trajectories are initialized from configurations in which the two N-terminal TMDs are already translated. To reduce computational cost, simulations are initiated with the second TMD pre-inserted in the lipid membrane (Fig 4A). The simulated sequences are limited to 90 CG beads in length, corresponding to a continuous stretch of amino acids starting from the second TMD (see S2 Appendix for all simulated sequences). Simulations are terminated when all CG beads of the H-segment either diffuse at least 2*σ* away from the translocon and span the membrane (integration, Fig 4A, S1 Movie) or when all CG beads have translocated to the lumenal side of the membrane (translocation, Fig 4A, S2 Movie). The probability of membrane integration is defined as the fraction of simulation trajectories that terminate by H-segment integration.

Fig 4B shows the comparison of the experimental versus the simulated probability of H-segment membrane integration as a function of the number of leucine residues in the H-segment. The results of the experimental assay [51] are plotted in black squares and the shaded region indicates outcomes within 1 kcal/mol of the experimental measurement as determined by a best fit of the apparent free energy of integration via a sigmoidal curve [51]. The calculated results from the 3D-CG model simulations are plotted in red circles. In agreement with the experiments, the 3D-CG model shows that H-segment integration increases with the number of leucines. Although slightly shifted to the right of the experimental curve, the simulation results recover the same sigmoidal dependence of integration on leucine content and are within 1 kcal/mol accuracy of the experiment [51]. These results indicate that the 3D-CG model correctly predicts trends in NC membrane integration using only information about the protein sequence as input.

Fig 4C and 4D investigate the issue of mapping from trios of amino-acid residues to a single CG bead. There are three possible CG representations (frameshifts) of the NC sequence that arise from the 3:1 mapping of amino-acid residues to CG beads as shown in Fig 4C. Since there is no basis for choosing any one frameshift over the other two, each of the possible frameshifts is simulated, and the calculated membrane integration probabilities shown in Fig 4B is the averaged value over all three frameshifts. For each frameshift and for each of the eight H-segment sequences, 100 trajectories are calculated (ranging from 20–3000 s in time) leading to 2,400 total simulations which required a total of 15,520 CPU hours on 2.6–2.7 GHz Intel Xeon processors. All CG bead sequences used in the simulations are provided in S2 Dataset. Fig 4D shows the membrane integration probability for the H-segment sequences for each individual frameshift. Results based on individual frameshifts are comparable, with a notable discrepancy for the 7 leucine H-segment in Frame 1 where the particular grouping of amino acids into triplets resulted in an H-segment for which the integration probability is relatively low. This sensitivity to the choice of triplets is addressed by simply averaging the results over all three frameshifts, which is done for the results in Fig 4B.

### Effect of single-residue mutations on the probability of membrane integration

As shown in Fig 4B, experiments and the 3D-CG model simulations both show that increasing the hydrophobicity of a H-segment by mutating alanine residues to leucine residues increases the probability of H-segment membrane integration. von Heijne and co-workers have extended this analysis by determining the effect of all twenty amino acids on the probability of H-segment membrane integration in the context of the Lep construct [51]. Assuming that there is an effective two-state equilibrium between the integration and translocation outcomes, the probability of integration can be converted into an apparent free energy of integration, Δ*G*_app_, defined by [51]

$$\Delta G_{\text{app}}=-kT\ln\left[p(\text{integration})/p(\text{secretion})\right].$$ (18)

By mutating the central residue of the H-segment in the same Lep construct used in the section *Probability of membrane integration for NC segments of varying hydrophobicity*, von Heijne and coworkers measured $\Delta G_{\text{app}}^{\text{aa}}$, or the single-residue apparent free energy of integration, for all twenty naturally occurring amino-acid residues, thus deriving a “biological hydrophobicity scale” in analogy to other hydrophobicity scales [63]. Calculating the probability of membrane integration of the same set of H-segments with the 3D-CG model provides a means to validate the predicted effect of single amino-acid residue mutations.

The simulation procedure for calculating the biological hydrophobicity scale is the same as illustrated in Fig 4A). To determine $\Delta G_{\text{app}}^{\text{aa}}$ for all 20 amino acids, 22 experimentally studied constructs of the mutated Lep sequence are mapped to a CG representation. Results are averaged over all three frameshifts for each of the 22 constructs, requiring a total of 66 CG bead sequences. All CG bead sequences modeled are provided in S2 Dataset. The probability of H-segment membrane integration is calculated from an ensemble of 200 trajectories (ranging from 20–2000 s in time) per sequence, leading to a total of 13,200 simulations which required a total of 77,003 CPU hours on 2.6–2.7 GHz Intel Xeon processors.

The probability of H-segment membrane integration is converted to a $\Delta G_{\text{app}}^{\text{aa}}$ following the procedure of von Heijne and coworkers described below [51]. The $\Delta G_{\text{app}}^{\text{aa}}$ for alanine and leucine are determined first from a linear fit of Δ*G*_app_ for H-segments with 3 to 7 Leucine residues from the simulated membrane integration probability curves (Fig 4B) using

$$\Delta G_{\text{app}}=n_{\text{Leu}}\;\left(\Delta G_{\text{app}}^{\text{Leu}}-\Delta G_{\text{app}}^{\text{Ala}}\right)+19\Delta G_{\text{app}}^{\text{Ala}}.$$ (19)

$\Delta G_{\text{app}}^{\text{aa}}$ for alanine and leucine are found to be 0.13 kcal/mol and -0.43 kcal/mol respectively. Experimentally determined values for alanine and leucine are 0.1 kcal/mol and -0.6 kcal/mol respectively. The difference in $\Delta G_{\text{app}}^{\text{aa}}$ between simulation and experiment for leucine gives rise to the slight rightward shift of the simulated membrane integration probability curve compared to the experiment in Fig 4B.

To obtain $\Delta G_{\text{app}}^{\text{aa}}$ for the remaining amino acids, we employ [51]

$$\Delta G_{\text{app}}^{\text{aa}}=\Delta G_{\text{app}}^{x[\text{aa}]x}-\Delta G_{\text{app}}^{x[\text{ref}]x}+\Delta G_{\text{app}}^{\text{ref}}.$$ (20)

Δ*G*^x[aa]x^ is the apparent free energy of integration for an H-segment construct with the probed amino acid (aa) at the midpoint of the H-segment Δ*G*^x[ref]x^ is the apparent free energy of integration for the same H-segment where the probed amino acid is replaced by a reference amino acid with a known apparent free energy of integration, $\Delta G_{\text{app}}^{\text{ref}}$. The reference amino acids employed match those used in Ref. [51] and are specified in S2 Dataset.

The H-segment constructs were chosen to have a leucine content such that the probability of membrane insertion for the sequence is nearly 50% to yield maximum sensitivity in the experimental assay [51]. For cysteine and methionine, we added two additional leucines to the simulated H-segment constructs compared to the experimental constructs to yield additional sensitivity in the computation.

**Fig 5:**
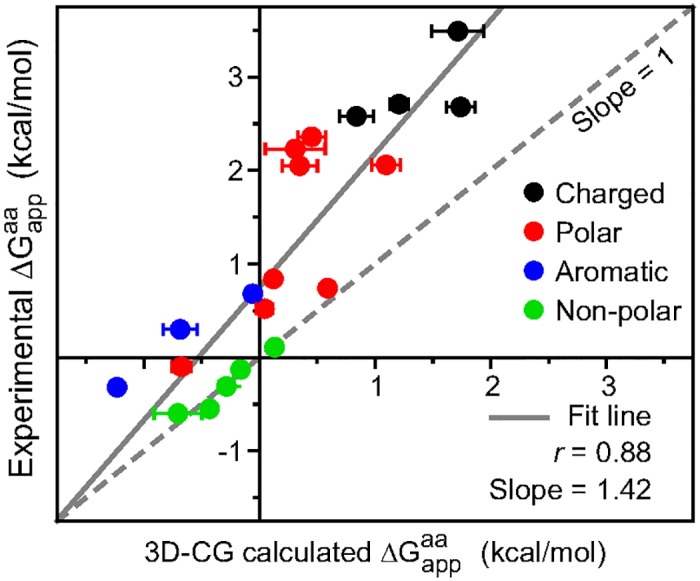
Experimental versus simulated predictions of the single-residue apparent free energy of integration. Each point corresponds to a different amino acid, with the character of the amino acid indicated by its plotted color. Each 3D-CG calculated $\Delta G_{\text{app}}^{\text{aa}}$ value the average of three frameshifts, the error bars indicate the standard error of the mean.

Fig 5 compares the values of $\Delta G_{\text{app}}^{\text{aa}}$ determined experimentally to the values of $\Delta G_{\text{app}}^{\text{aa}}$ calculated using the 3D-CG model. Each point represents a single amino acid. Points are colored by grouping amino-acid residues as charged (black), polar (red), aromatic (blue), or non-polar (green). The solid line is a linear fit to the data, while the dashed line illustrates a perfect correlation as a guide to the eye. Each $\Delta G_{\text{app}}^{\text{aa}}$ value is calculated from the average of three frameshifts (defined as in Fig 4). The average standard deviation between the frameshift results is 0.2 kcal/mol, the error bars indicate the standard error of the mean. Individual frameshift values are reported in Table S3 in S3 Appendix. The experimental and 3D-CG simulation scales are highly correlated (*r* = 0.88), confirming that the 3D-CG model reproduces trends in $\Delta G_{\text{app}}^{\text{aa}}$ with high fidelity. The data points largely lie above the dashed line, indicating that the 3D-CG simulations slightly overestimate the experimentally observed degree of integration. These results thus indicate that the 3D-CG is capable of reproducing the effect of single-residue mutations in good agreement with available biophysical measurements, although the quantitative agreement with experiments may still be improved via further model refinements.

### Kinetic regulation of TMD topology

**Fig 6:**
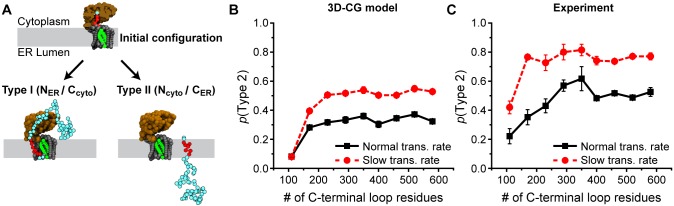
3D-CG model predictions for TM topology. (A) Snapshots of the initial system configuration and the two possible TM topologies. (B) 3D-CG model simulation results showing the fraction of trajectories that reach the Type 2 topology as a function of the number of C-terminal loop residues, plotted for a normal translational rate (solid black) and a slowed translation rate (dashed red). (C) Experimental results from Göder et al [14], with a normal translation rate (solid black) and with the addition of cyclohexamide, a translation rate inhibitor (dashed red).

In addition to determining whether NC segments integrate into the membrane as TM domains, the translocon regulates the orientation with which TM segments integrate (Fig 6A) [14, 33, 75]. In particular, Spiess and co-workers found that an engineered TM signal anchor (H1Δ22) integrates in either the N_ER_/C_cyt_ (i.e. Type 1) or the N_cyt_/C_ER_ (i.e. Type 2) topology; it was also found that decreasing the rate of ribosomal translation by adding cycloheximide increases the preference for the Type 2 topology [14]. Furthermore, increasing the length of the soluble loop flanking the C-terminus of the TM segment also increases the probability that the TM segment obtains the Type 2 topology until the probability eventually plateaus for a sufficiently long loop length. Previous work using the 2D-CG model qualitatively captured both these trends and revealed that the mechanistic basis for the kinetic effect is flipping of the NC from the Type 1 topology to the Type 2 topology as a function of time [43]. However, due to the lack of residue-specific interactions in the 2D-CG model, this work employed model sequences. Additionally, due to the simplified geometric representation of the 2D-CG model, it predicted that *p*(Type 2) plateaus at shorter C-terminal lengths than is observed in the experiments. While the 2D-CG model can provide mechanistic insights [43], quantitative agreement with the experiments is poor compared to the 3D-CG model when directly mapping the amino-acid sequence (Fig S2 of S3 Appendix and corresponding discussion). Here, we test the 3D-CG model for predicting TMD topogenesis and the effect of translation kinetics on topology.

The simulation approach for modeling TMD topogenesis is summarized in Fig 6A (see S3 and S4 Movies for example trajectories). The H1Δ22 sequence is mapped to CG beads, and the results are averaged over all three frameshifts. Nine different lengths of the C-terminal soluble loop are mapped directly from the experimental constructs used in [14]. The first 99 residues of the sequence are assumed to be part of helical domains based on secondary structure predictions from the PSIPRED server [73, 76]. Simulations are initialized from configurations in which four CG beads are translated and have not yet entered the translocon. Simulations are terminated when CG beads of the TMD have all integrated into the lipid bilayer in either an Type 1 or Type 2 topology and diffuse 10*σ* away from the translocon. The final TMD topology is determined from the position of the C-terminal CG bead relative to the membrane upon simulation termination (Fig 6B). All simulations are performed with either the default translation rate of 5 residues/second (fast translation) or with a reduced translation rate of 1.25 residues/second (slow translation) to model the effect of adding cyclohexamide in the experimental assay. 200 trajectories (ranging from 25–1200 s in time) are simulated for each of the three frameshifts and for each of the nine loop lengths and at both translation rates, leading to a total of 10,800 trajectories which required a total of 149,009 CPU hours on 2.6–2.7GHz Intel Xeon processors.

Fig 6B compares the simulated and experimental results for the probability with which the TMD obtains the Type 2 topology as a function of the length of the C-terminal soluble loop. The results of the experimental assay are plotted on the right for reference. Results for the normal translation rate are in solid black lines, while results for the reduced translation rate are in dashed red lines. The simulation results correctly reproduce the trends observed in the experiments, including the increased probability of the Type 2 topology for longer C-terminal loop lengths and the eventual plateau in the probability of the Type 2 topology at long C-terminal loop lengths. Furthermore, like the experimental results, the CG model predicts a significant shift to greater Type 2 integration upon reducing the rate of ribosomal translation.

### Conclusions

We present a refined CG model for co-translational membrane protein integration via the Sec translocon that captures the detailed three-dimensional geometry of the ribosome-translocon complex from high-resolution structural data [6, 25] and that describes residue-specific interactions between the NC and translocon based on detailed MD simulations. The bottom-up parameterization approach utilized here employs extensive residue-based coarse-grained simulations to inform the model parameters without the need for additional experimental inputs. In this work, the 3D-CG model is applied to calculate the membrane integration efficiency and topology of TMDs, where the only required input is the amino-acid sequence and NC secondary structure. The 3D-CG model captures the experimentally observed [51] sigmoidal dependence of the probability of TMD integration on substrate hydrophobicity. We extend this analysis to study the effect of all twenty amino-acids on the membrane integration probability yielding values of residue-specific TMD membrane integration probabilities in good agreement with the experimentally observed “biological hydrophobicity” scale [51]. These results demonstrate that the 3D-CG model successfully combines factors that are known from previous work to affect TMD integration at the translocon, such as interactions of the nascent chain and the translocon channel interior [37, 38, 40], the non-equilibrium nature of peptide elongation [37, 43], and the sequence context of the TMD [77]. This suggests that the 3D-CG model is well suited for future applications to investigate phenomena such as the experimentally observed position dependence of the biological hydrophobicity scale [35] and the dependence of the observed hydrophobicity values on the amino-acid residues flanking the TMD [77]. The specific interactions between the NC and the translocon, determined as part of this study, already suggest a mechanism by which flanking residues can affect TMD integration; the high barrier for the translocation of charged residues limits translocation, resulting in more integration. Finally, the 3D-CG model accurately describes the experimentally observed effect of translation rate and C-terminal loop length on TMD topogenesis [14]. The 3D representation of the model ensures the correct ribosome-translocon geometry and volume scaling behavior necessary to capture the C-terminal length dependence of TMD topology, an effect not captured in a previous 2D model [40].

The main advantage of the 3D-CG model presented here, compared to previous work, is that it requires few assumptions. NC properties are directly mapped from the underlying amino acid sequence, the ribosome/translocon geometry is mapped from available structural data, and there is no projection onto a two-dimensional subspace. Provided only with an amino acid sequence and a secondary structure assignment, the 3D-CG model obtains striking agreement with experiment, validating the ability of the 3D-CG model to predict key aspects of Sec-fascilitated protein translocation and membrane integration.

We additionally emphasize that the 3D-CG model provides a refinable framework for simulating IMP co-translational membrane integration via the Sec translocon. Currently, the bottom-up parameterization strategy uses MARTINI PMFs for four distinct tripeptide substrates as input information. The 3D-CG model parameterization could be refined, either by calculating the PMF of other substrates using the MARTINI force field, by considering the role of changes in substrate protonation state in the channel interior, or by calculating PMFs using an atomistic force field. Furthermore, improved methods for parameterization and uncertainty quantification can be employed to determine parameter sets consistent with the available data [78]. All of these refinements can be made within the current 3D-CG model framework, and they will enable incorporation of additional information and improved quantitative prediction. This framework can also be naturally extended to include additional complexity, such as NC secondary and tertiary structure, other proteins that are part of the Sec translocon complex, and a heterogeneous translation rate. Future studies aimed at the prediction of multispanning IMP topology will guide model development.

The 3D-CG model presented here broadens the capability of computer simulation approaches for future studies of the TMD membrane insertion process. In particular, by providing residue-specific NC-translocon interactions the current model enables direct comparison to biophysical measurements of forces between the NC and the translocon due to hydrophobic and electrostatic forces [34, 52]. Furthermore, the realistic representation of the structure and interactions enables future mutational studies and comparison of species-specific features of the ribosome-translocon complex to obtain a detailed understanding of key residues that impact TMD integration and topogenesis. The encouraging agreement between 3D-CG model simulation outcome and experiments for single-spanning TMDs displays the capabilities of the 3D-CG framework. It enables the calculation of minute-timescale trajectories in three dimensions, facilitating computational studies that are not possible using existing models with less detail, or existing models that are unable to reach the biologically relevant timescales. The 3D-CG model, with initial model parameters obtained here using a bottom-up strategy, provides a systematically improvable framework for the simulation of co-translational membrane protein integration via the Sec translocon.

## Supporting information

S1 Appendix MARTINI simulation details. (PDF)

S2 Appendix Determination of ribosome and translocon coordinates. (PDF)

S3 Appendix Additional CG model details. (PDF)

S1 Movie Illustrative trajectory for H-segment membrane integration. A single, representative 3D-CG trajectory for the process described in Fig 4A, leading to the H-segment integration product. Simulations are initiated with the TMD preceding the H-segment inserted in the lipid membrane. The total simulation time corresponds to 20 s.
(MP4)

S2 Movie Illustrative trajectory for H-segment translocation. A single, representative 3D-CG trajectory for the process described in Fig 4A, leading to the H-segment translocation product. Simulations are initiated with the TMD preceding the H-segment inserted in the lipid membrane. The total simulation time corresponds to 20 s.
(MP4)

S3 Movie Illustrative trajectory for Type 1 signal sequence membrane insertion. A single, representative 3D-CG trajectory for the process described in Fig 6A, leading to the Type 1 product. The simulation is of a signal sequence with C-terminal length of 170 amino acids, the total simulation time corresponds to 38 s.
(MP4)

S4 Movie Illustrative trajectory for Type 2 signal sequence membrane insertion. A single, representative 3D-CG trajectory for the process described in Fig 6A, leading to the Type 2 product. The simulation is of a signal sequence with C-terminal length of 170 amino acids, the total simulation time corresponds to 83 s.
(MP4)

S1 Dataset Channel coordinates. (XLSX)

S2 Dataset List of sequences simulated. (XLSX)
