# Supplementary material for: Structurally detailed coarse-grained model for Sec-facilitated co-translational protein translocation and membrane integration
Source: PLoS Comput Biol. 2017 Mar 22;13(3):e1005427. doi: 10.1371/journal.pcbi.1005427 (PMC5381951; doi:10.1371/journal.pcbi.1005427)
Supplement: S1 Appendix — (PDF) [file pcbi.1005427.s001.pdf]

S1 Appendix MARTINI simulation details

MARTINI simulation initialization and equilibration

In this section, we describe the initialization of simulations from Main Text section *Residue-based coarse-grained simulations*. Residue-based coarse-grained (RBCG) simulations of the translocon are set up using GROMACS 4.5 [1]. The initial system is prepared by converting the crystal structure of the α, β, and γ-subunits of the Archaeal Sec-translocon (PDB ID: 1RHZ) to a MARTINI RBCG representation using the martinize.py script [2]. Scaffolding interactions are introduced to correctly preserve protein tertiary structure [3].Scaffolding interactions are included for a pair of CG particles if both are contained in one of the following subsets of the translocon: (*i*) residues Lys^{2}-Val^{45} and Ile^{71}-Pro^{205} in the α-subunit, and the entire β-subunit; (*ii*) residues Trp^{29}-Lys^{66} in the γ-subunit; and (*iii*) residues Pro^{205}-Leu^{433} in the α-subunit. Scaffolding interactions are also included between particles in subsets *i* and *ii*, and between particles in subsets *ii* and *iii*. Scaffolding interactions are only included between CG beads that are separated by 5-9 Å in the original mapping from the crystal structure, and that do not already share a bonded interaction. Scaffolding interactions between pairs of CG particles are weak harmonic distance restraints with an equilibrium distance equal to the distance in the original crystal structure mapping and a force constant equal to 100 kJ mol^{-1} nm^{-2}.

The RBCG translocon is oriented with respect to a pre-equilibrated lipid bilayer consisting of 400 POPC lipid molecules using the Lambada package [4]. The lipids are then packed around the translocon using the inflategro2 package [4], and lipids that clash with the translocon are removed from the simulation. CG water molecules are added using the *genbox* command in GROMACS, and ions are added using the *genion* command in GROMACS to reach charge neutrality and a physiological salt concentration ($\sim$50 mM). The final system contains the translocon, 368 POPC molecules, 6209 CG water molecules, 6 sodium ions, and 17 chloride ions.

The entire system is equilibrated in the MARTINIv2.2 force field using the following protocol; (*i*) 50 steps of steepest descent energy minimization, (*ii*) a 20 ps NPT simulation at 310 K and 1 bar with 2 fs timesteps, and (*iii*) a 100 ns NPT simulation at 310 K and 1 bar with 20 fs timesteps. During steps *ii* and *iii* protein backbone CG beads are position restrained during the equilibration using harmonic constraints with a force constant of 1000 kJ mol^{-1} nm^{-2}. Both NPT simulations use the leap-frog integrator, Berendsen temperature coupling using a temperature coupling constant, τ_{T}, of 0.5 ps, and semi-isotropic pressure coupling using the Berendsen barostat with a pressure coupling constant, τ_{p}, of 1.2 ps and an isothermal compressibility of 4.5 x 10^{-5} bar^{-1}.

For simulations with tripeptide substrates, MARTINI representations of NC substrates are added to the system and overlapping water molecules were removed. The new system with the substrate is then equilibrated again using the three step equilibration cycle as described above.

Collective variables used in MARTINI simulations

Here, we describe the collective variables from Main Text section *Residue-based coarse-grained simulations*. Three collective variables (CVs) are used in the MARTINI simulations. This section lists the CVs and provides details on any biasing force applied to these CVs.

The first CV, $d_{\mathrm{LG}}(\mathbf{r})$, describes the opening of the translocon lateral gate (LG). It is defined as the minimum distance between the CG backbone beads in TM2b (Ile^{75}-Gly^{92}) and TM7 (Ile^{257}-Arg^{278}) in the translocon α-subunit (Fig. S1A). Specifically, $d_{\mathrm{LG}}(\mathbf{r})$ is expressed as

$$d_{\mathrm{LG}}(\mathbf{r}) = \frac{\alpha}{\ln\left[\sum_{ij}\exp(\alpha/|r_{ij}|)\right]}, \tag{1}$$

where the sum is over all pairs i, j for which i is a backbone CG bead in TM2b and j is a backbone CG bead in TM7, $|r_{ij}|$ is the distance between CG bead i and CG bead j and α is a large number, the value for α is chosen depending on the distance at which the $d_{\mathrm{LG}}(\mathbf{r})$ is constrained, to avoid precision errors in evaluating the exponential. For simulations of the translocon in the closed LG conformation, a harmonic constraint is placed on $d_{\mathrm{LG}}(\mathbf{r})$ with equilibrium distance 0.7 nm, force constant 2000 kJ mol^{-1} nm^{-2}, and $\alpha = 250$. For

simulations of the translocon in the open LG conformation, a harmonic restraint is placed on $d_{\mathrm{LG}}(\mathbf{r})$ with equilibrium distance 1.4 nm, force constant 2000 kJ mol^{-1} nm^{-2}, and $\alpha = 500$.

The second CV, $d_z(\mathbf{r})$, describes the position of the NC substrate along the translocon channel axis, perpendicular to the lipid bilayer. It is the dot product of a distance vector, v_{s}, between the geometric center of the NC substrate and the geometric center of CG beads describing the translocon pore residues (Ile^{75}, Val^{79}, Ile^{170}, Ile^{174}, Ile^{260}, and Leu^{406}) (red vector in Fig. S1B), and a normal vector, v_{c}, in the positive z-direction originating from the geometric center of CG beads describing the translocon pore residues (blue vector in Fig. S1B). For the umbrella-sampling trajectories used to construct the PMFs for NC substrate translocation along the channel axis, a harmonic restraint is placed on $d_z(\mathbf{r})$, the equilibrium distance, $d_{z,0}$, and force constant, κ_z, used is listed in the description of the relevant simulations (Table S1).

The third CV, $d_{xy}(\mathbf{r})$, describes the distance between the NC substrate and the translocon channel axis in the plane parallel to the lipid bilayer. A soft-wall potential is used to ensure that the NC substrate does not diffuse far from the translocon (Fig. S1C). The potential is expressed as

$$U_{\mathrm{wall}}(\mathbf{r}) = \left\{ \begin{array}{ccc} \kappa_{\mathrm{w}}(d_{xy}(\mathbf{r}) - d_{\mathrm{w}})^2 & , & d_{xy}(\mathbf{r}) > d_{\mathrm{w}} \\ 0 & , & d_{xy}(\mathbf{r}) \leq d_{\mathrm{w}} \end{array} \right\}, \tag{2}$$

where the force constant, κ_{w}, is set to 2000 kJ mol^{-1} nm^{-2}, and the wall distance, d_{w}, is set to 1.2 nm.

All collective variables used in the MARTINI simulations were implemented using PLUMED version 2 [5].

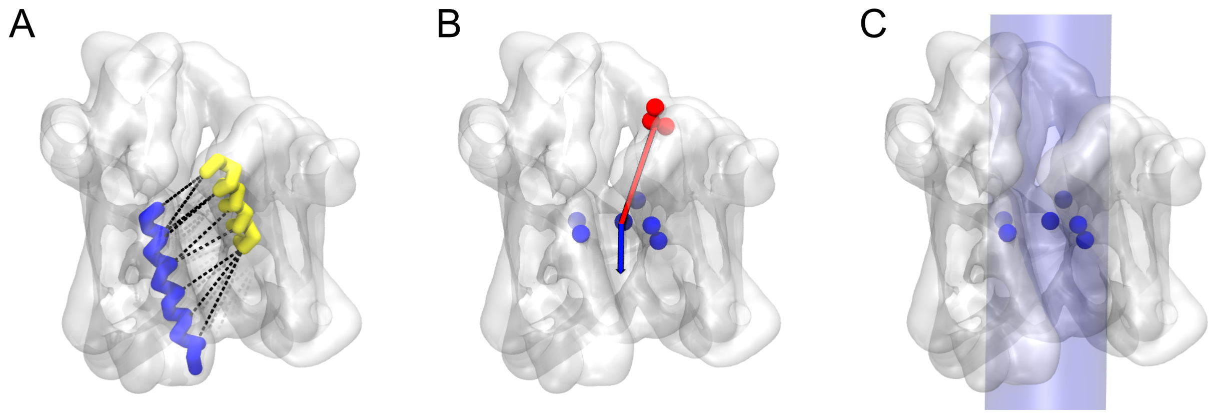

Fig S1. Visual representation of the collective variables used in the MARTINI simulations. (A) The minimum distance between the backbone CG beads in TM2b (yellow) and TM7 (blue) defines the conformational state of the translocon lateral gate. (B) The position of the NC substrate along the channel axis is calculated as the dot product between the NC-pore vector (red) and the channel-axis vector (blue). (C) The radial distance of the NC substrate to the channel axis is constrained to be inside a 1.2 nm cylinder (blue region). In all panels the translocon is shown as a white transparent surface.

Details on MARTINI simulations for translocation PMF profiles

In this section, we describe the calculation of the translocation PMF profiles from Main Text section *Residue-based coarse-grained simulations*. MARTINI simulations are performed using a Langevin dynamics integrator with a 20 fs timestep. Lennard-Jones interactions are shifted from 0.9 to 1.2 nm. Electrostatic interactions are calculated using the smooth Particle Mesh Ewald (PME) method with a grid spacing of 0.12 nm and a short-range cutoff of 1.2 nm. The dielectric constant is set to 2.5 as recommended for the MARTINI polarizable water model. The simulation temperature is maintained at 310 K using a Langevin dynamics integrator. The simulation pressure is maintained at 1 bar via a semi-isotropic Parrinello-Rahman barostat with a coupling time constant of 12 ps and an isothermal compressibility of 3 x 10^{-4} bar^{-1}. These parameters

follow recent recommendations for optimal MARTINI simulations using the polarizable water molecule and PME electrostatics [6].

To fully sample the PMF along the channel axis, 51-64 umbrella-sampling trajectories are performed for each tri-peptide substrate in which $d_{z,0}$ is restrained (simulations summarized in Table S1). Collective variables were restrained as described in the section *Collective variables used in MARTINI simulations*, and simulations were carried out in both the open and closed channel conformation. For each simulation reported, at least 100 ns of equilibration is performed followed by 400 ns which is sampled for the calculation of the PMF. Translocation PMFs are obtained from the umbrella-sampling trajectories using the Weighted Histogram Analysis Method [7].

Convergence of MARTINI simulations

We assess the convergence of the MARTINI simulations described in the Main Text section *Residue-based coarse-grained simulations* by plotting the PMF as a function of increasing sampling time Fig. S2 and observe that all PMFs have converged with respect to simulation time. We also plot the overlap in umbrella sampling windows in Fig. S3 and observe sufficient overlap between all windows.

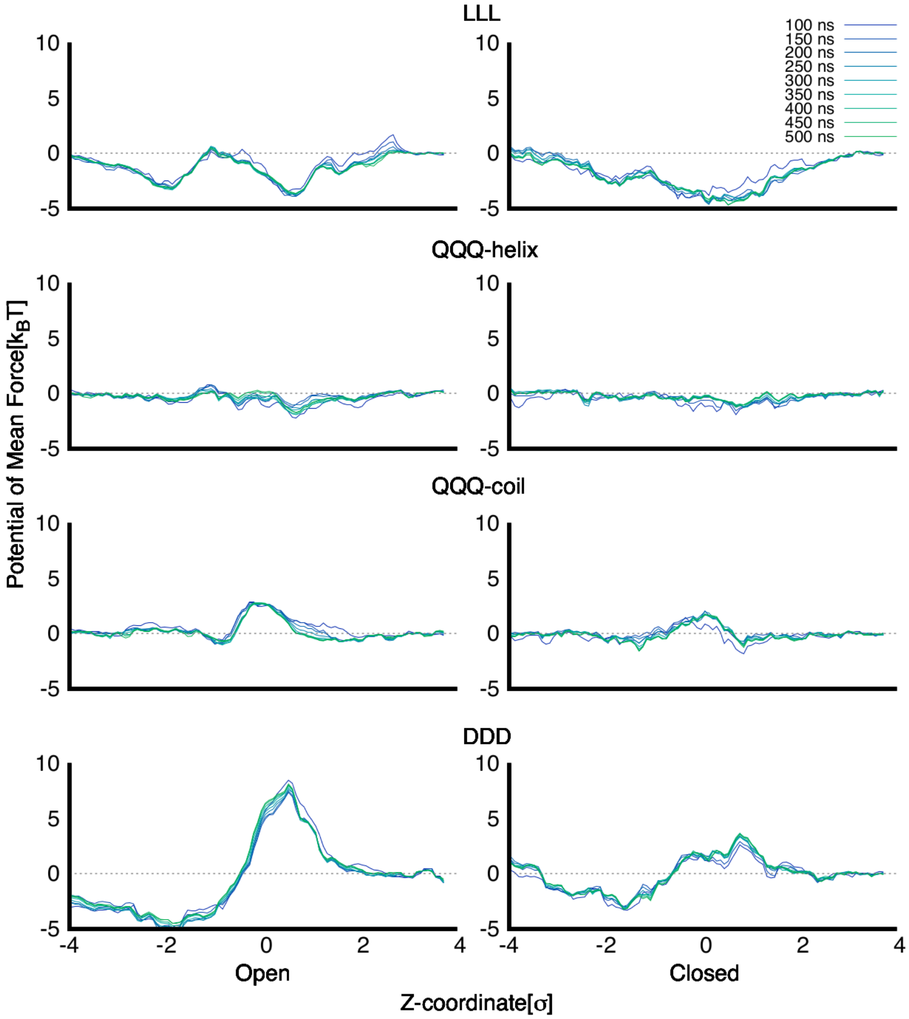

Fig S2. Convergence of MARTINI PMFs as the sampling time increases. The PMF is calculated after 10 ns, 50 ns, 100 ns, 150 ns, 200 ns, 250 ns, 300 ns, 350 ns and 400 ns of sampling time and plotted as a gradient of blue to green lines. The similarity in calculated PMFs as the simulation time increases is used to assess convergence.

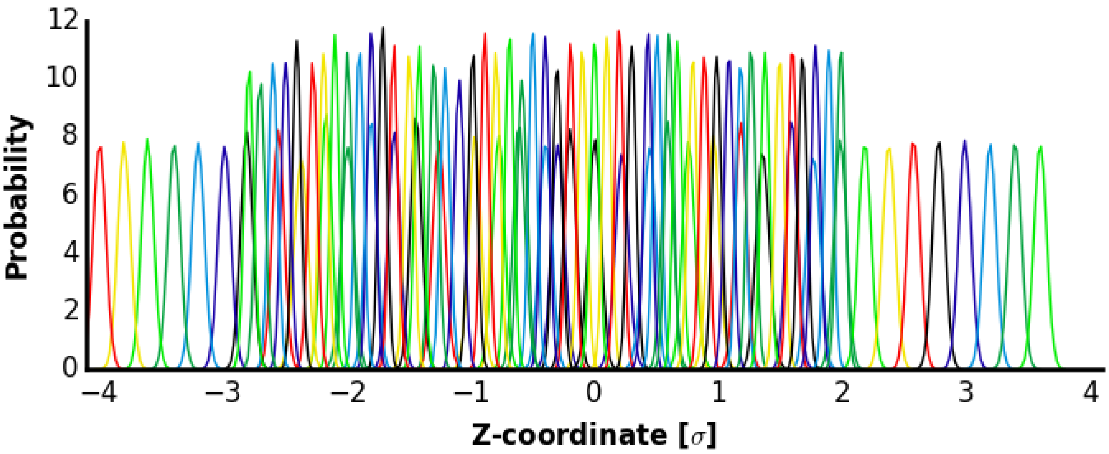

Fig S3. Overlap in umbrella sampling windows for the LLL substrate in the open channel. Additional windows with stiffer springs are added to improve overlap between z = -3σ and z = 2σ

Substrate	LG state	$d_{z,0}$ [nm] range	Spacing	Equilibration time [ns]	κ_z [kJ mol^{-1} nm^{-2}]
LLL	closed	-4.0 — -1.8	0.2	100	1000
LLL	closed	-1.6 — 1.4	0.1	100	2000
LLL	closed	1.6 — 3.6	0.2	100	1000
LLL	open	-4.0 — -3.0	0.2	600	1000
LLL	open	-2.8 — 2.0	0.1	600	2000
LLL	open	2.2 — 3.6	0.2	600	1000
QQQ_{helix}	closed	-4.0 — -3.0	0.2	100	1000
QQQ_{helix}	closed	-2.8 — -1.4	0.1	100	2000
QQQ_{helix}	closed	-1.3 — 0.8	0.1	600	2000
QQQ_{helix}	closed	0.9 — 1.4	0.1	600	2000
QQQ_{helix}	closed	1.6 — 2.0	0.2	600	1000
QQQ_{helix}	closed	2.2 — 3.6	0.2	100	1000
QQQ_{helix}	open	-4.0 — -3.0	0.2	600	1000
QQQ_{helix}	open	-2.8 — 2.2	0.1	600	2000
QQQ_{helix}	open	2.4 — 3.6	0.2	600	1000
QQQ_{coil}	closed	-4.0 — -3.0	0.2	100	1000
QQQ_{coil}	closed	-2.8 — -1.4	0.1	100	2000
QQQ_{coil}	closed	-1.3 — 1.0	0.1	600	2000
QQQ_{coil}	closed	1.1 — 2.0	0.1	100	2000
QQQ_{coil}	closed	2.2 — 3.6	0.2	100	1000
QQQ_{coil}	open	-4.0 — -2.4	0.2	600	1000
QQQ_{coil}	open	-2.2 — 2.2	0.1	600	2000
QQQ_{coil}	open	2.4 — 3.6	0.2	600	1000
DDD	closed	-4.0 — -1.8	0.2	100	1000
DDD	closed	-1.6 — 1.0	0.1	100	2000
DDD	closed	1.2 — 3.6	0.2	100	1000
DDD	open	-4.0 — -2.4	0.2	600	1000
DDD	open	-2.2 — 1.0	0.1	600	2000
DDD	open	1.2 — 3.6	0.2	600	1000

Table S1. Summary of MARTINI simulations used for translocation PMF construction.

References

[1] Pronk S, Páll S, Schulz R, Larsson P, Bjelkmar P, Apostolov R, et al. GROMACS 4.5: a high-throughput and highly parallel open source molecular simulation toolkit. Bioinformatics. 2013;29:845–854.

[2] de Jong DH, Singh G, Bennett WFD, Arnarez C, Wassenaar TA, Schäfer LV, et al. Improved Parameters for the Martini Coarse-Grained Protein Force Field. J Chem Theory Comput. 2013;9:687–697.

[3] Zhang B, Miller TF. Hydrophobically stabilized open state for the lateral gate of the Sec translocon. Proc Natl Acad Sci. 2010;107(12):5399–5404.

[4] Schmidt TH, Kandt C. LAMBADA and InflateGRO2: efficient membrane alignment and insertion of membrane proteins for molecular dynamics simulations. J Chem Inf Model. 2012;52(10):2657–2669.

[5] Tribello GA, Bonomi M, Branduardi D, Camilloni C, Bussi G. PLUMED 2: New feathers for an old bird. Comput Phys Commun. 2014;185(2):604–613.

[6] de Jong DH, Baoukina S, Ingólfsson HI, Marrink SJ. Martini straight: Boosting performance using a shorter cutoff and GPUs. Comp Phys Comm. 2016;199:1–7.

[7] Kumar S, Rosenberg JM, Bouzida D, Swendsen RH, Kollman PA. The weighted histogram analysis method for free-energy calculations on biomolecules. I. The method. J Comp Chem. 1992;13(8):1011–1021.
