# Supplementary material for: Structurally detailed coarse-grained model for Sec-facilitated co-translational protein translocation and membrane integration
Source: PLoS Comput Biol. 2017 Mar 22;13(3):e1005427. doi: 10.1371/journal.pcbi.1005427 (PMC5381951; doi:10.1371/journal.pcbi.1005427)
Supplement: S2 Appendix — (PDF) [file pcbi.1005427.s002.pdf]

S2 Appendix Determination of ribosome and translocon coordinates

Translocon CG bead coordinates

In this section, we describe the determination of translocon channel CG bead coordinates from Main Text section *3D-CG Model Geometry.* Channel coordinates are obtained from equilibrium MARTINI simulations of the Sec-translocon in explicit solvent. Simulations are set up as described in the section *MARTINI simulation initialization and equilibration*, with a alpha-helical poly-leucine (L_{30}) substrate inside the channel. The distance between the geometric center of the substrate and the geometric center of the translocon pore residues is restrained about zero using a harmonic potential with force constant 200 kJ mol^{-1} nm^{-2}.

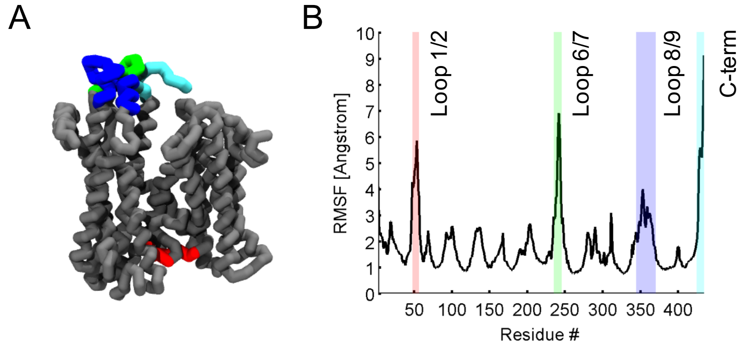

Fig S1. Flexible loops identified by equilibrium MARTINI simulations. (A) Representative snapshot from a MARTINI simulation of the translocon in the open conformation. Flexible loops are colored as follows; Thr^{47}-Phe^{56} red, Pro^{234}-Gly^{245} green, Lys^{343}-Arg^{369} blue, and Val^{423}-Lys^{433} cyan. Non-protein atoms are not shown for clarity. (B) Root-mean squared fluctuation (RMSF) per residue backbone bead calculated from 500 ns of equilibrium MARTINI simulation on the Sec-translocon in the absence of a substrate. Flexible loop regions are highlighted, with colors as in part (A).

To obtain coordinates for the channel in the closed conformation, the system is simulated for 500 ns with $d_{\mathrm{LG}}(\mathbf{r}) = 0.7$ nm Similarly, to obtain coordinates for the channel in the open conformation the system is simulated for 500 ns with $d_{\mathrm{LG}}(\mathbf{r}) = 1.4$ nm. The average Sec-translocon protein backbone coordinates over the simulation are mapped into the 3D-CG model using the mapping procedure as described in the Main Text section *3D-CG Model Geometry.* Flexible regions of the channel, defined as loop regions with a high RMSF measured from equilibrium MARTINI simulations (Fig S1), are excluded from the mapping. An additional bead is included to represent the stable salt bridge between K26 and E421.

Ribosome CG bead coordinates

In this section, we describe the determination of ribosome CG bead coordinates from Main Text section *3D-CG Model Geometry.* The geometry of the ribosome is obtained by mapping the ribosome-translocon complex from a recent high-resolution cryo-EM structure [1] (PDB ID: 3J7Q) onto CG beads. Because CG bead coordinates for the translocon are obtained from the equilibrated average coordinates of residue-based coarse-grained simulations in the absence of the ribosome, the CG model ribosome must be aligned with the CG model translocon by the procedure described below (shown in Fig S2). The ribosome-translocon cryo-EM structure [1], which contains a translocon in a partially cracked conformation similar to the closed conformation, is aligned with the CG model of the translocon in the closed conformation by minimizing the root mean squared deviation between the LG helices in the CG model translocon and the LG helices mapped from the cryo-EM structure. After alignment, only CG beads of the ribosome that are within 9σ of origin and with 5σ of the channel axis are explicitly retained as CG beads in the final simulation system.

In total, the ribosome consists of 357 CG beads. Complete coordinate files for the CG representation of the ribosome-translocon complex with the translocon in both conformations are included in S1 Dataset.

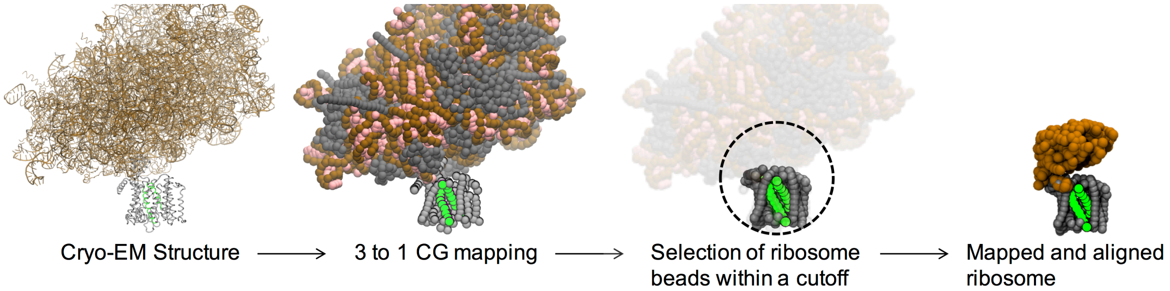

Fig S2. Mapping and alignment of ribosome in 3D-CG model. The starting coordinates of the mammalian ribosome-translocon complex from the Voorhees *et al.* cryo-EM structure are shown in the stick representation. These coordinates are mapped in place onto CG beads with CG beads from protein residues shown in gray, CG beads associated with the RNA backbone shown in brown and CG beads associated with RNA nucleobases shown in pink. The lateral gate CG beads, shown in green, are aligned with the equilibrated translocon CG beads from MARTINI simulations and ribosome beads within 9σ of the origin are retained for the final model.

Translocon CG bead charges

To avoid the assignment of large net charges to the Sec translocon CG beads via the 3:1 mapping protocol, channel beads were assigned a +1 charge if the net charge of the underlying amino acids was positive and a -1 charge if the net charge of the underlying amino acids was negative. In contrast, for nascent chain beads the charge of the CG bead is the sum of the charge of the underlying amino-acid residues. This was done because the CG representation of the translocon was not simulated with all possible frameshifts, as was done for the nascent chain. Only four translocon beads are affected by this issue, and all of these beads are located on the channel exterior. As a result, the PMF for the translocation of charged residues in the 3D-CG model is not sensitive to this subtlety related to determining the charge of the translocon CG beads (Fig S3).

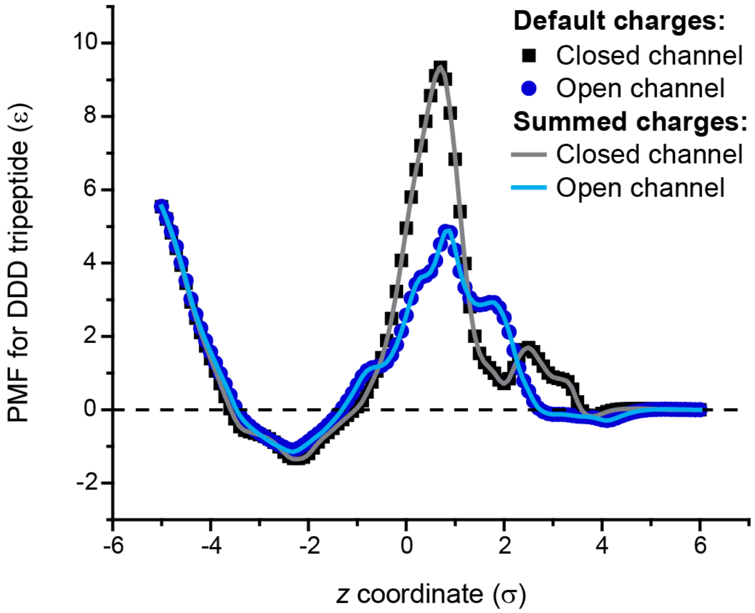

Fig S3. Comparison of the PMF obtained with translocon bead charges as described in the main text (symbols), or with translocon bead charges equal to the sum of the charge of the underlying amino-acid residues (lines). No discernible difference is observed.

References

[1] Voorhees RM, Fernández IS, Scheres SHW, Hegde RS. Structure of the mammalian ribosome-Sec61 complex to 3.4 A resolution. Cell. 2014;157(7):1632–1643.
