# Supplementary material for: Structurally detailed coarse-grained model for Sec-facilitated co-translational protein translocation and membrane integration
Source: PLoS Comput Biol. 2017 Mar 22;13(3):e1005427. doi: 10.1371/journal.pcbi.1005427 (PMC5381951; doi:10.1371/journal.pcbi.1005427)
Supplement: S3 Appendix — (PDF) [file pcbi.1005427.s003.pdf]

S3 Appendix Additional CG model details

Tabulation of the NC-translocon potential energy surface for computational efficiency

To increase the computational efficiency of the 3D-CG model and to exploit the static nature of the translocon and ribosome CG beads (i.e., non-NC CG beads), interactions between NC and non-NC CG beads are tabulated. At the start of a 3D-CG model trajectory, potential energy tables are generated for these interactions. The tables store interaction energies on a rectangular grid surrounding the translocon/ribosome beads. The grid size is chosen such that there is a distance of at least the non-bonded interaction cut-off length between the grid edge and any translocon/ribosome bead. With a grid-spacing of 0.1σ this yields 121x122x115-element tables. Separate tables are generated for Lennard-Jones and electrostatic interactions. All simulations and analytical potentials of mean force for the 3D-CG model described in this work utilize these potential energy tables.

From the potential energy tables, the interaction energy for a NC bead with the translocon/ribosome beads at any point in Cartesian space is obtained via tri-linear interpolation of the potential energy at the nearest eight grid-points. Specifically,

$$\begin{aligned} U(\mathbf{r}) &= \bar{x}\bar{y}\bar{z}U_{111} + \bar{x}\bar{y}(1-\bar{z})U_{110} + \bar{x}(1-\bar{y})\bar{z}U_{101} \\ &+ \bar{x}(1-\bar{y})(1-\bar{z})U_{100} + (1-\bar{x})\bar{y}\bar{z}U_{011} + (1-\bar{x})\bar{y}(1-\bar{z})U_{010} \\ &+ (1-\bar{x})(1-\bar{y})\bar{z}U_{001} + (1-\bar{x})(1-\bar{y})(1-\bar{z})U_{000}, \end{aligned} \tag{1}$$

where the local coordinate, $\bar{x} = (x - x_0)/(x_1 - x_0)$; with x the x-coordinate of the NC bead, x_1 the x-coordinate of the nearest grid-points with $x_1 > x$, and x_0 the x-coordinate of the nearest grid-points with $x_0 < x$. Potential energy values at the grid-points are labeled using the local coordinates at the grid-point, $U_{\bar{x}\bar{y}\bar{z}}$.

Forces on a NC bead at any point in Cartesian space are similarly obtained using interpolation. For example, for the force in the x-direction is given by,

$$\begin{aligned} F_x(\mathbf{r}) &= [U_{011} - U_{111}]\bar{y}\bar{z} \\ &+ [U_{010} - U_{110}]\bar{y}(1-\bar{z}) \\ &+ [U_{001} - U_{101}](1-\bar{y})\bar{z} \\ &+ [U_{000} - U_{100}](1-\bar{y})(1-\bar{z}). \end{aligned} \tag{2}$$

Amino Acid	WW TFE (kcal/mol)	WW TFE (epsilon)
A	0.5	0.81
C	-0.02	-0.03
D	3.64	5.91
E	3.63	5.89
F	-1.71	-2.78
G	1.15	1.87
H	2.33	3.78
I	-1.12	-1.82
K	2.8	4.55
L	-1.25	-2.03
M	-0.67	-1.09
N	0.85	1.38
P	0.14	0.23
Q	0.77	1.25
R	1.81	2.94
S	0.46	0.75
T	0.25	0.41
V	-0.46	-0.75
W	-2.09	-3.39
Y	-0.71	-1.15

Table S1. Wimley-White water-octanol transfer free energy values for all twenty naturally occurring amino acids

Reduced units in the 3D-CG model

Simulations with the 3D-CG model use Lennard-Jones reduced units, which are described in this section. As discussed in the main text, the length-scale is set by the Kuhn-length of a polypeptide chain, $\sigma = 0.8$ nm, and the energy unit is $\epsilon = 1k_BT$. The diffusion coefficient used in the 3D-CG simulations is based on previous work [1,2]. It is reduced by 3-fold compared to the value used in 2D-CG simulations to account for the faster translocation times in three-dimensions, $\tau_{tr} \propto \frac{R_g^2}{D}$. The reduced mass unit, $m^* = 300$ Da, is the approximate mass of a CG bead containing three amino acid residues. From this, one can derive the reduced time-unit in the 3D-CG simulations,

$$t^* = \sigma\sqrt{\frac{m^*}{\epsilon}}s, \quad (3)$$

which yields $t^* \approx 0.01$ ns, the same value as used in the previously published 2D-CG model [2]. This reduced time unit is used to compare simulation results to experimental data. Although caution should be taken when relating CG timescales with real time, the agreement between 3D-CG simulations and experiments probing kinetic effects on TMD integration (Main Text Fig 6) are encouraging.

Robustness to simulation timestep

Table S2 presents a test of the robustness of the reported results with respect to the timestep employed in the 3D-CG simulations. In addition to the results obtained using the 300 ns timestep employed throughout the main text, results were recomputed using a timestep of 150 ns. The probability for membrane integration, reported in Main Text Fig 4B, is recomputed for H-segments containing 4 and 5 Leucine residues. The probability of Type 2 topology for a H1Δ22 signal anchor, reported in Main Text Fig 6B, is recomputed for sequences with a C-terminal length, L, of 400 residues. In all cases, the results obtained with a 150 ns timestep are within error of the results presented in the main text.

Experiment	Δt=300 ns	Δt=150 ns
Membrane integration, 4 Leu	26% ± 5%	27 % ± 7%
Membrane integration, 5 Leu	70% ± 3%	70% ± 2%
Type 2 topology, L=400 residues, 5 res/sec	40% ± 2%	40 % ± 5%
Type 2 topology, L=400 residues, 1.25 res/sec	66% ± 2%	64% ± 2%

Table S2. Comparison of results with different simulation time steps

Channel CG bead type assignment and fitting MARTINI PMFs

Here, we describe the fitting of the Martini PMFs described in Main Text section *Parameterization of NC-Translocon interactions*. The MARTINI PMFs are fit by defining two bead types for the translocon channel; one "normal" bead type, and one "confined" bead type, that have distinct parameter values. When fitting the PMF for translocating a LLL substrate across the closed and open channel, the "confined" bead type are assigned as beads for which $0.3 < z < 1.0$ and $x^2 + y^2 < 2.25$. All other beads are assigned the "normal" bead type. These bead type assignments are shown in Fig S1 with normal bead types shown in gray and confined bead types shown in red. When fitting the PMF for translocating a DDD substrate across the closed and open channel the "confined" bead type are assigned as those beads for which $-0.1 < z < 1.1$ and $x^2 + y^2 < 4.8$. These bead type assignments are shown in Fig S1 with normal bead types shown in gray and "confined" bead types as the set of both red and pink beads.

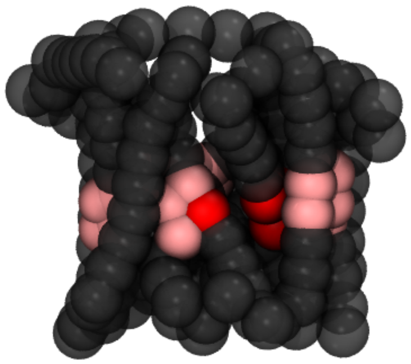

Fig S1. Channel Bead Assignment. Bead assignments in the closed channel conformation with the "default" bead types shown in gray, the repulsive confined beads are shown in red spheres and the attractive confined beads are both the red and pink spheres.

We obtain the values of ϵ_j^{int} using the lmfit Python module to perform a weighted least squares fit between $-2 < z < 5$ where the absolute error is exponentially weighted to prioritize fitting the peaks and valleys. The region $z < -2$ is not used for the fit because this region contains contributions from flexible loop regions of the translocon in the MARTINI PMF simulations; these loop regions have been removed in the 3D-CG model and therefore features in this region are not expected to be captured in the 3D-CG model. In order to match the MARTINI residue-based coarse-grained simulations for which the plug domain has been removed and the ribosome is not present, the CG beads corresponding to the ribosome and the plug domain are removed during the fitting procedure. For the 3D-CG calculations the plug domain beads are assumed to have normal bead type interactions.

Effect of single-residue mutations on membrane integration by frameshift

Residue	Frameshift 1 ΔG_{app}	Frameshift 2 ΔG_{app}	Frameshift 3 ΔG_{app}	Average ΔG_{app}	Std. err
I	-0.99	-0.82	-0.30	-0.70	0.21
F	-1.28	-1.26	-1.16	-1.23	0.04
V	-0.41	-0.26	-0.18	-0.28	0.07
C	-0.23	-0.16	-0.10	-0.16	0.04
M	-0.71	-0.79	-0.52	-0.67	0.08
W	-0.85	-0.40	-0.80	-0.68	0.14
T	-0.05	0.18	0.00	0.04	0.07
Y	0.05	-0.21	-0.01	-0.06	0.06
G	0.56	0.54	0.67	0.59	0.04
S	0.14	0.08	0.14	0.12	0.02
N	0.13	0.64	0.27	0.35	0.15
H	1.29	1.03	0.97	1.10	0.12
P	0.02	0.36	0.56	0.31	0.26
Q	0.61	0.39	0.36	0.45	0.12
K	1.46	1.03	1.13	1.21	0.08
R	0.73	1.00	0.79	0.84	0.14
E	1.88	1.70	1.65	1.74	0.13
D	2.00	1.71	1.44	1.72	0.22

Table S3. ΔG_{app} values for each residue, separated by frameshift. The result presented in main text Figure 5 is the average over the three possible frameshifts. All values are presented in kcal/mol.

Comparison with the 2D-CG model

In Fig S2, we present a comparison of the probability of a H1Δ22 signal anchor obtaining the Type 2 topology calculated using the 3D-CG model and the 2D-CG model. As in Fig 6 of the Main Text, the probability of Type 2 topology, p(Type 2), is plotted as a function of the C-terminal length, L, for two different translation rates. In the 2D-CG model, the normal translation rate corresponds to 24 residues/second and the slow translation rate corresponds to 6 residues/second.

Simulations performed using the 2D-CG model utilize a direct 3:1 mapping procedure to assign the hydrophobicity and charge of each CG bead, replicating the procedure used in the 3D-CG model. For each of the three frameshifts, 100 trajectories are simulated. This differs from our previous study of topogenesis using the 2D-CG model [2], in which the amino-acid sequence of the experimental construct was not directly mapped to a CG representation; instead, in the earlier work, each construct was represented as a "model" sequence in which CG beads in the C-terminal loop were assigned uniform hydrophilic transfer free energies, CG beads in the transmembrane domain were assigned a uniform hydrophobic transfer free energy, and charges were added to CG beads to approximate the charge distribution of the experimental construct. This previous work demonstrated that the 2D-CG model qualitatively captures the effect of C-terminal length and translation rate on the probability of Type 2 topology for the model sequences. Here, we compare the 2D-CG model versus the 3D-CG model for sequences that are directly mapped from the underlying amino-acid sequence (i.e., not model sequences).

As seen in Fig S2, when the CG beads are directly mapped from the amino-acid sequence, the 3D-CG model both qualitatively and quantitatively outperforms the 2D-CG model by more accurately capturing the probability of Type 2 topology and correctly demonstrating the experimentally observed trends. Specifically, the probability of Type 2 insertion plateaus for long C-terminal domain lengths and the probability of Type 2 insertion increases with decreasing translation rate (Fig S2). In comparison, the 2D-CG model fails to capture either trend and significantly underestimates the probability of Type 2 insertion. We emphasize that this failure is not inherent to the 2D-CG model itself, since application with model sequences yields these qualitative trends [2]. Nonetheless, these results indicate that the 3D-CG model improves significantly over the 2D-CG model in applications involving the direct mapping of the amino-acid sequence.

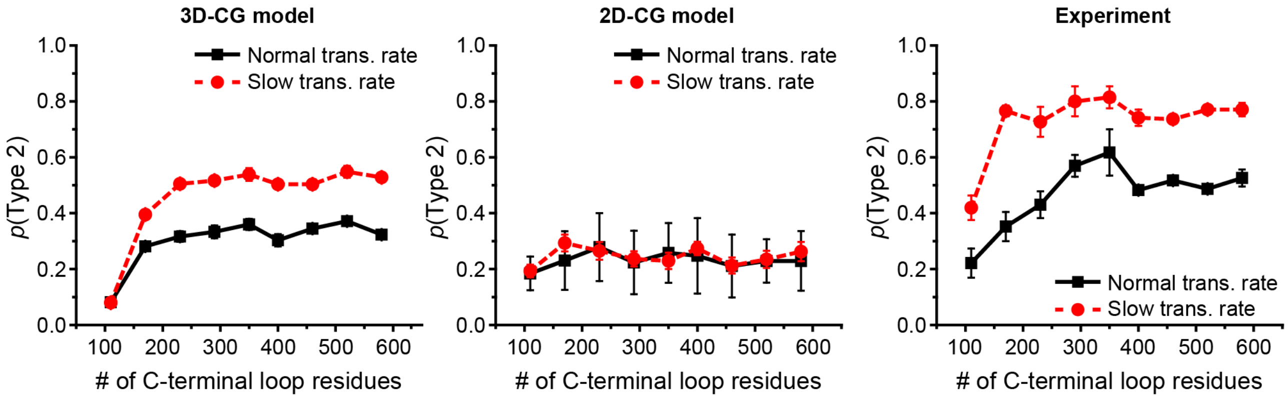

Fig S2. Comparison of 3D-CG model simulation results and 2D-CG simulation results showing the fraction of trajectories that reach the Type 2 topology as a function of the number of C-terminal loop residues, plotted for a normal translational rate (solid black) and a slowed translation rate (dashed red). Data for the 3D-CG model and the experiment are reproduced from Main Text Fig 6.

References

[1] Zhang B, Miller TF. Direct simulation of early-stage sec-facilitated protein translocation. J Am Chem Soc. 2012;134(33):13700–13707.

[2] Zhang B, Miller TF. Long-Timescale Dynamics and Regulation of Sec-Facilitated Protein Translocation. Cell Rep. 2012;2(4):927–937.
